# Species-Specific Structural Requirements of Alpha-Branched Trehalose Diester Mincle Agonists

**DOI:** 10.3389/fimmu.2019.00338

**Published:** 2019-02-28

**Authors:** Alyson J. Smith, Shannon M. Miller, Cassandra Buhl, Robert Child, Margaret Whitacre, Roman Schoener, George Ettenger, David Burkhart, Kendal Ryter, Jay T. Evans

**Affiliations:** ^1^Center for Translational Medicine, Missoula, MT, United States; ^2^Division of Biological Sciences, Missoula, MT, United States; ^3^Department of Biomedical and Pharmaceutical Sciences, Missoula, MT, United States; ^4^Department of Chemistry, University of Montana, Missoula, MT, United States

**Keywords:** mincle receptor, trehalose diester, *Mycobacterium tuberculosis*, C type lectin receptor, adjuvant, Th17

## Abstract

Despite the ever present need for an effective *Mycobacterium tuberculosis* (*Mtb*) vaccine, efforts for development have been largely unsuccessful. Correlates of immune protection against *Mtb* are not wholly defined, but Th1 and likely Th17 adaptive immune responses have been demonstrated to be necessary for vaccine-mediated protection. Unfortunately, no approved adjuvants are able to drive a Th17 response, though recent clinical trials with CAF01 have demonstrated proof of concept. Herein we present the discovery and characterization of a new class of potential Th17-inducing vaccine adjuvants, alpha-branched trehalose diester molecules (αTDE). Based off the *Mtb* immunostimulatory component trehalose dimycolate (TDM), we synthesized and evaluated the immunostimulatory capacity of a library of structural derivatives. We evaluated the structure activity relationship of the compounds in relation to chain length and engagement of the Mincle receptor, production of innate cytokines from human and murine cells, and a pro-Th17 cytokine profile from primary human peripheral blood mononuclear cells. Murine cells displayed more structural tolerance, engaging and responding to a wide array of compound chain lengths. Interestingly, human cells displayed a unique specificity for ester chains between 5 and 14 carbons for maximal immune stimulating activity. Evaluation of two distinct αTDEs, B16 and B42, in concert with a recombinant *Mtb* antigen demonstrated their ability to augment a Th17 immune response against a *Mtb* antigen *in vivo*. Collectively this data describes the species-specific structural requirements for maximal human activity of alpha-branched trehalose diester compounds and demonstrates their capacity to serve as potent Th17-inducing adjuvants.

## Introduction

Globally, there are still a number of pathogens that cause a significant burden of disease for which limited to no vaccines or prophylaxis is available. Among these is the gram negative bacterium *Mycobacterium tuberculosis* (*Mtb*) which infects an estimated 10 million people per year resulting in over a million deaths annually. Of even greater concern is the increasing spread of extensively drug-resistant strains (XDR), which result in over 0.5 million deaths per year. It was recently determined that the majority of cases of XDR tuberculosis are likely due to transmission rather than inadequate treatment (1), suggesting that control of the epidemic of drug-resistant tuberculosis would benefit greatly from a new safe and effective vaccine. However, vaccine development against this pathogen has been plagued by ineffective candidates and the absence of strong clinical correlates of protection.

While the understanding of protective immunity to *Mtb* remains incomplete, animal model and clinical data suggest that a functional CD4+ T cell response featuring the expression of Th1-type cytokines is crucial for protection against TB (2–4). Recent data also support a role for IL-17 in the protective immunity conferred by an anti-*Mtb* vaccine. For instance, upon aerosol infection with Mtb, murine lungs show increased expression of IL-17, which is ablated in the absence of IL-23 (5). Pre-clinical observations (5–7) suggest that the IL-23/IL-17 axis alone is not sufficient for protecting from *Mtb* infection but that it can augment the immune response directed against *Mtb*. Th17 offers long-term control of bacteria growth, a critical component of an effective vaccine (8, 9). While Th17 cells induce factors (e.g., IL-17, IL-8) involved in neutrophil recruitment at the site of infection, they are also shown to be responsible for recruiting IFNγ-producing protective memory CD4+ Th1 cells to the site of *Mtb* infection (10). Khader and colleagues demonstrated that a successful protective vaccine strategy will rely on populating the bacterial entry areas with IL-17-expressing cells that will respond rapidly to the infection.

Thus, a successful *Mtb* vaccine must be able to induce these adaptive immune responses to confer protection. Vaccine strategies which target immunostimulatory components of the pathogen itself are often the most successful at simulating durable immunity following vaccination. The cell wall of *Mtb* is structurally composed of many immunostimulatory lipids and systematic analysis of these components resulted in identification of the immunostimulatory glycolipid trehalose-6,6′-dimycolate (TDM) (11). Nearly fifty years later, the C type lectin receptor (CLR) Mincle was identified as the innate immune receptor responsible for the activity of TDM (12, 13). Because of its highly potent immune-stimulating properties, TDM has proven too reactogenic for human use (14, 15); however, many groups have explored synthetic derivatives of this natural molecule with matched immunostimulatory activity but diminished toxicity. The most advanced of these synthetic derivatives is the non-branched, synthetic diester compound trehalose dibehenate (TDB). For clinical use, TDB has been formulated with dimethyldiocyadecylammonium (DDA) in a nanoparticle liposome formulation, CAF01 (16). This adjuvant formulation has been evaluated in human clinical trials with various vaccine candidates including HIV, influenza and TB (16–19) with varied success.

Previous studies have described the structure-activity-relationship (SAR) of synthetic trehalose derivatives in an effort to explore the underlying structural drivers of biological activity and reactogenicity. Some of these studies have focused on understanding the impact of the carbohydrate moiety (20, 21) or acyl chain length on activity of unbranched trehalose diesters (TDEs). In these early SAR studies, longer acyl chains (between 20 and 26 carbons) linked to the trehalose core were necessary for activity of these compounds (22, 23). Subsequently, trehalose *di*esters demonstrated superior innate immune stimulating activity when compared to *mono*ester counterpoints but failed to demonstrate significant differences in activity between compounds with differing acyl chain lengths (24). However, this subsequent study focused on unbranched acyl chains between 14 and 22 carbons long so questions remain as to the activity of the shorter acyl chain and branched acyl chain containing compounds. Furthermore, assessment of acyl chain length of symmetrical α-branched TDEs between 18 and 34 carbons did not establish a clear correlation between length and biological response (25).

These initial studies have paved the way for improved understanding of this class of synthetic TDM-derivatives but systematic biological evaluation of TDE acyl chain variants and SAR in both human and mouse cells is still lacking. Therefore, in this study we created an exhaustive library of synthetic α-branched trehalose diester compounds (αTDEs) with varying chain lengths and assessed their ability to modulate both human and murine immune responses. Striking species-specific differences in SAR were noted between human and murine systems. We determined that in human cells an optimal chain length for maximal immune stimulation is between 5 and 14 carbons, while the murine Mincle receptor was more promiscuous responding robustly to branched TDEs with chain lengths of 10–22. We also demonstrate that the most active compound is an effective adjuvant with recombinant *Mtb* antigen, M72, to drive an antigen-specific Th17 immune response *in vivo*. Collectively this study highlights the importance of SAR in the immunostimulatory activity of novel synthetic adjuvants in both mouse and human cells and demonstrates the superior adjuvanticity of mid-chain length αTDE compounds to drive a vaccine-mediated Th17 immune response.

## Materials and Methods

### Materials

THP-1 and Raw264.7 cells were obtained from ATCC (Manassas, VA), TDM was obtained from Sigma (St. Louis, MO), TDB from Avanti Polar Lipids (Alabaster, AL). MMG was prepared according to the procedure of Andersen et al. (26). αTDEs were synthesized from 2,2′,3,3′,4,4′-hexa-O-trimethyl silyl-α,α'-D-trehalose and the appropriate carboxylic acid potassium salt as previously described (27). The general procedure for preparation of alpha branched TDEs was completed as follows:

2,2′,3,3′,4,4′-Hexa-trimethylsilyl-α,α-D-trehalose (2.1767 g, 2.81 mmol) was dissolved in anhydrous methylene chloride (32 mL) and anhydrous pyridine (1.8 mL, 22.28 mmol) under nitrogen with stirring before being placed on a salted ice bath. Triflic anhydride (1 mL, 5.92 mmol) was added over 30 min maintaining the internal temperature below 6°C. The reaction was monitored by TLC (20% ethyl acetate/heptane) and upon completion was diluted with methylene chloride (100 mL). The solution was washed twice with cold 0.5 M HCl (50 mL), aqueous saturated sodium bicarbonate, brine, dried over sodium sulfate, filtered and concentrated providing the 2,2′,3,3′,4,4′-hexa-trimethylsilyl-6,6′-bis(triflate)-α,α-D-trehalose (2.8845, 99%) which can be used as is or stored in a −80°C freezer for 4–6 weeks.

Alpha branched acid (2 mmol) was dissolved in anhydrous THF (2 mL) and treated with 2M potassium trimethylsilanolate in THF (1.1 mL, 2.20 mmol). If a precipitate formed, the acid salt was filtered and used as is. If not the reaction was poured into diethyl ether the resulting precipitate was isolated by filtration. The solid was dried under high vacuum to provide the potassium salt of the alpha branched acid and was combined with 2,2′,3,3′,4,4′-hexa-trimethylsilyl-6,6′-bis(triflate)-α,α-D-trehalose (933.7 mg, 899 μmol) and 18-crown-6 (237.4 mg, 899 μmol) in anhydrous toluene (17 mL). The reaction was heated to 70°C and until the reaction was complete by TLC (30% ethyl acetate/heptane). Heptane (30 mL) was added and the organic solution was washed with water (20 mL) and concentrated. The resulting residue was subjected to chromatography on silica gel eluting with a gradient of 0–25% ethyl acetate in heptane. All product fractions were combined, reduced in vacuo and dried under high vacuum to provide 2,2′,3,3′,4,4′-hexa-trimethylsilyl-6,6′-bis(acyl)-α,α-D-trehalose.

2,2′,3,3′,4,4′-hexa-trimethylsilyl-6,6′-bis(acyl)-α,α-D-trehalose product was dissolved in anhydrous 1:1 methanol/methylene chloride (0.1 M) and Dowex 50WX8 (500 mg per 250 μmol) was added with stirring and monitored by TLC (30% ethyl acetate/heptane for starting material and 15% methanol in methylene chloride for product). Upon completion, the reaction was filtered through celite concentrated and purified by chromatography on silica gel eluting with a 0–50% methanol in methylene chloride gradient.

All compounds were isolated to at least 95% purity and fully characterized by ^1^H NMR and HRMS (Supplemental Figure 1 and data not shown).

All compounds were solubilized and diluted in a 50% isopropanol (IPA)/50% Isooctane solution. For plate coating, 20 μl of compounds were applied to the bottom of tissue culture plates and allowed to dry for > 1 h for solvent evaporation. Compounds for *in vivo* studies were formulated in DDA:DSPC (distearoyl-sn-glycero-3-phosphocholine) liposomes using an adapted thin-film method. Briefly, lipid components were dissolved in 9:1 chloroform:methanol in a round bottom flask and evaporated by rotary evaporation under vacuum to create a lipid thin film. The lipid thin films were dried under high vacuum overnight at room temperature to remove any residual organic solvent. Films were rehydrated using 10 mM TRIS buffer (pH 7.4) at a lipid concentration of 8.4 mg/ml and sonicated to reduce particle size at 66°C. Samples were sonicated until the particle size (Z_avg_) was below 100 nm as measured by dynamic light scattering (Supplemental Figure 2) and were then sterile filtered by syringe 0.22 μm PVDF filtration into sealed, sterile, depyrogenated glass vials. Quantitation of TDE compound in liposomal formulations was determined by RP-HPLC using gradient elution with charged aerosol detection against a 5-point standard curve.

### Transgenic HEK Cell SEAP Assays

Human and mouse Mincle expressing HEK cells were obtained from Invivogen (San Diego, CA). Cells were cultured according to the manufactures instructions in DMEM with 10% FBS, 50 U/ml penicillin, 50 mg/ml streptomycin, 100 mg/ml Normocin, 2 mM L-glutamine, 30 μg/ml blasticidin, 1 μg/ml puromycin and 1x HEK-Blue™ CLR Selection. For assay, indicated compounds were serially diluted in diluent (50%IPA/50% isooctane), 20 μl of a 10x final concentration were applied to the bottom of a 96-well tissue culture plate and the solvent was evaporated for >1 h in a biosafety hood. HEK cells were applied to the plates at a density of 3 × 10^5^ cells/well and incubated for 18–24 h at 37°C. Cell supernatants were harvested and analyzed via the manufacturer's instructions using Hek-Blue^TM^ Detection. SEAP activity was assessed by reading the optical density (OD) at 620–655 nm with a microplate reader; data are expressed as the fold change in OD over vehicle treated cells.

### Human Cell Isolation and Compound Treatment

PBMCs were obtained via leukapheresis from normal donors (AllCells, Berkeley, CA) or fresh blood was obtained from health human donors through a University of Montana Institutional Review Board (IRB) approved protocol. PBMCs obtained via leukapheresis were washed in 1x PBS and cryopreserved in freezing media (50% RPMI, 40% FCS, 10% DMSO) for future use. PBMCs from on-site human donors were separated from whole blood via density gradient separation using Histopaque 1077 (Sigma). Cells were washed in RPMI media containing 10% heat inactivated FBS, penicillin/streptomycin/glutamine (complete media) and resuspended at the desired cell concentration in complete media. Cells were treated with the indicated compound concentrations by addition to plates coated with a serial dilution of the stock compound in diluent (50% isopropanol/50% isooctane, fully evaporated).

### Cytokine Analysis

Supernatants were harvested from treated cells following 18–24 h of incubation. Supernatants were analyzed using a DuoSet TNFα ELISA (R&D Systems, Minneapolis, MN) or Luminex multiplex panel for analytes TNFα, IL-1β, IL-6, IFNγ, IL-12p70, and IL-23 (R&D Systems) per the manufacturer's instructions. Multiplex analysis was performed using a Luminex 200 instrument (Luminex Corporation) and analyzed with StarStation2.3 software. ELISAs were read on a plate reader at 450 nm and cytokine concentration was calculated by fitting the standard curve OD values to a 4-parameter logistical model using curve fitting software (XLfit, IDBS, Alameda, CA).

### *In vivo* Experiments

Animal studies were carried out in an OLAW and AAALAC accredited vivarium in accordance with University of Montana's IACUC guidelines for the care and use of laboratory animals. Groups of 10–14 BALB/c mice were vaccinated intramuscularly with 0.125 μg M72 antigen and indicated concentrations of various CLR adjuvant candidates (liposomal formulations) in 50 μl total volume per injection. After 14 days, blood and serum samples were collected and a secondary vaccination was administered. At day 19 (5 days post-secondary vaccination), 3–6 mice per group were euthanized and spleens were harvested the assessment of cell-mediated immunity. At day 28 (14 days post booster vaccination) the remaining 7 mice in each group were euthanized and blood was collected for the measurement of M72 specific humoral immunity.

### ELISA for Anti-M72 Antibody Quantification

Sera was collected from mice 14 days post-primary and 14 days post-secondary injection and diluted according to the expected antibody response (between 1:10 and 1:5000). Plates were coated with 100 μl of the full-length M72 protein at 1 μg/mL. Following washing (PBS plus tween 20) and blocking (SuperBlock, Scytek Laboratories), plates were incubated with diluted serum for 1 h followed by anti-mouse IgG, IgG1, or IgG2a-HRP secondary antibody (Bethyl Laboratories) and TMB substrate (BD). Plates were read at 450 nm. Antibody titers were determined by calculating titer of each sample at OD 0.3.

### Splenocyte Restimulation and Cell-Mediated Immunity Analysis

Spleens were harvested from vaccinated mice 5 days after secondary injections and splenocytes were harvested by disruption of the spleens through a 100 μm filter. Red blood cells were lysed by incubation with red blood cell lysis buffer (Sigma) for 5 min followed by washing in 1x PBS. Cells were plated in a 96 well plate at 5 × 10^6^ cells/well in 200 μL complete RPMI1640 media. Cells were incubated with 1 μg/mL M72 antigen for 6 h at 37°C. After 7 h, GolgiPlug (Brefeldin A, BD Biosciences) was added to each well and incubated at 37°C for a further 12 h. Following incubation, cells were stained with the cell surface antibodies against CD3e PerCP-Cy5.5 (Tonbo Biosciences, 145-2C11), CD4a APC-Cy7 (Tonbo Biosciences, RM4-5) and CD8a PE-Cy7 (Tonbo Biosciences, 53–6.7) and viability stain (Ghost 510, Tonbo Biosciences). Cells were treated with cytofix/cytoperm (BD) and stained with anti-IFNγ PE-CF594 (BD, XMG1.2), anti-IL2 FITC (Biolegend, JES6-5H4), anti-IL-5 PE (BD, TRFK5), anti-IL17A BV421 (Biolegend, TC11-18H10.1), and anti-TNFα APC (Invitrogen, MP6-XT22). Data was collected using an LSRII flow cytometer (BD) and analyzed using FlowJo 10.0 software (TreeStar). Splenocytes were gated on live, single cells, then CD3+ to identify all T cells, then gated as CD4+CD8- to identify CD4 T cells or CD4-CD8+ to identify CD8 T cells. Gates for intracellular cytokine positive CD4 or CD8 T cells are set based on re-stimulation of naïve splenocytes. Gating strategies for the detection of antigen specific CD4+ and CD8+ T cells are outlined in Supplemental Figure 3.

## Results

### Activation of Mincle in Response to a Library of α-Branched TDEs

**Figure 1:**
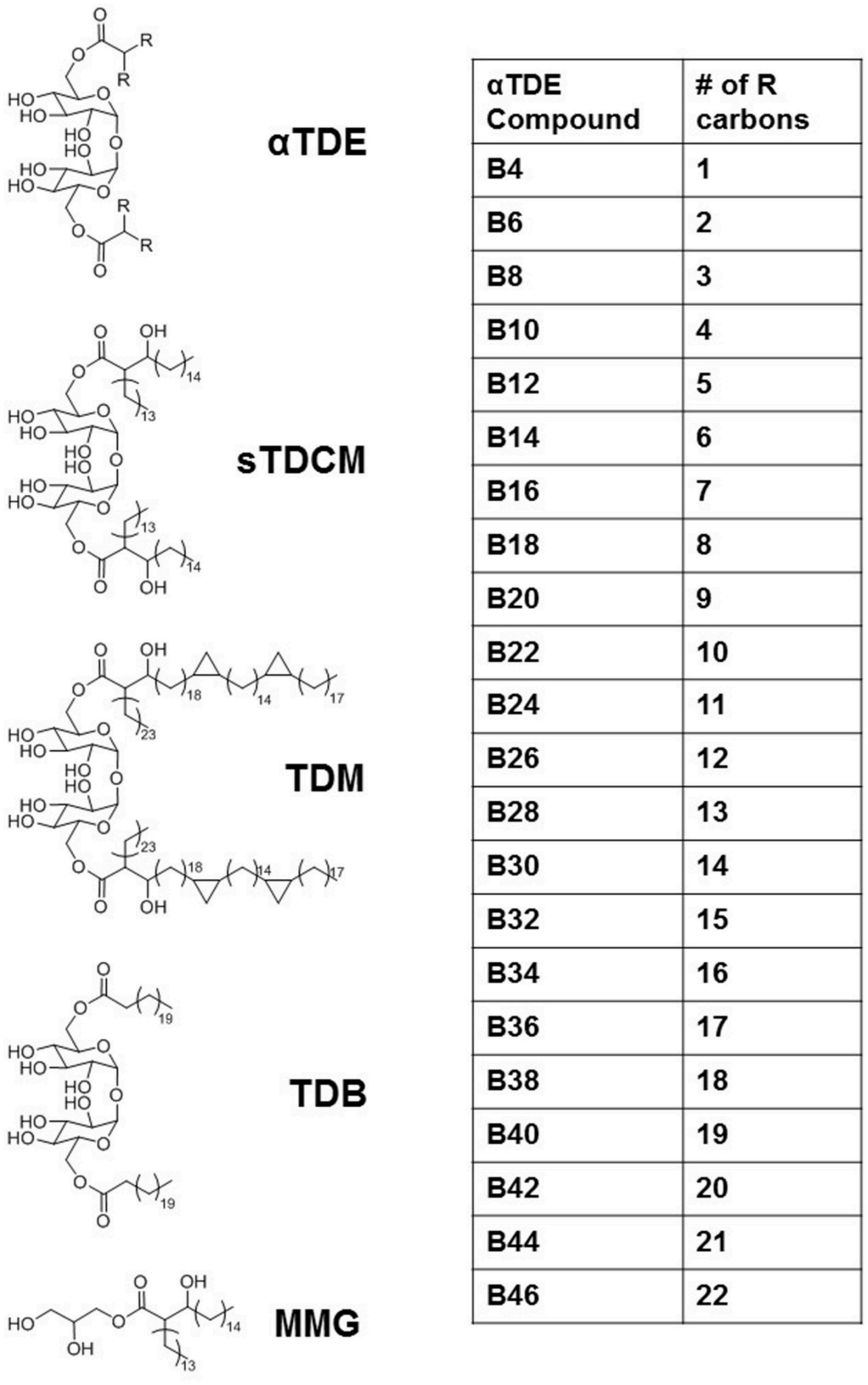
Overview of structures of αTDE-derivative compounds and controls.

The Mycobacterium extract trehalose dimycolate (TDM) has been identified as an immunostimulant that works primarily through activation of the pattern recognition receptor Mincle (12, 13). It has widely been shown to induce inflammatory cytokine production, such as TNFα, and promote the development of a Th17 immune response through the induction of innate cytokines such as IL-6, IL-1β, and IL-23. We developed a library of synthetic alpha-branched diester trehalose derivatives (αTDEs) (Figure 1) to evaluate the structure activity relationship of these compounds. In addition, we comprehensively evaluated the immune stimulating capacity of these synthetic TDEs in both murine and human systems. This library of compounds ranged from a single carbon on each acyl-chain branch to lengths roughly equivalent to the shorter asymmetrical chain on TDM, 22 carbons.

**Figure 2:**
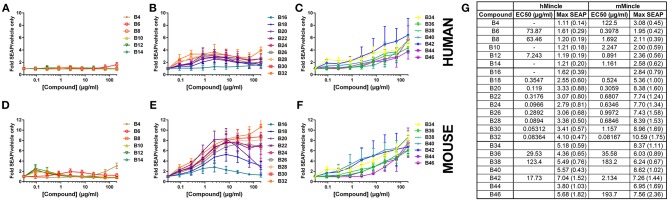
Activation of human or mouse Mincle in response to synthetic αTDE compounds. The indicated compounds were dissolved in 50% isopropanol/isooctane, serially diluted in vehicle and then dried to the bottom of a tissue culture plate. HEK cells transfected with human **(A–C)** or mouse **(D–F)** Mincle and an NF-κB-driven SEAP reporter were incubated with the compounds for 24 h followed by assessment of the supernatants for SEAP levels. Data are represented as fold change in OD_650_ over vehicle treated cells. Graphs are mean values from three independent experiments ± SEM. Table in **(G)** represents the average calculated EC50 and average max SEAP value (± SD, *n* = 3) for each compound in each cell line.

To investigate the capacity of these molecules to bind to and activate the Mincle receptor, we used human embryonic kidney (HEK) cells expressing the human or mouse Mincle receptor along with an NF-κB-driven secreted embryonic alkaline phosphatase (SEAP) reporter. Many of the αTDE compounds were able to induce production of SEAP in a dose dependent manner (Figure 2). HEK null cells (HEK cells containing the NF-kB reporter without Mincle receptor) were used as a negative controls to confirm the receptor specificity of the compounds in HEK cells expressing human or mouse Mincle reporter (Supplemental Figure 4). The positive control reference compounds, including synthetic trehalose dicorynomycolate (sTDCM), trehalose dibehenate (TDB) and TDM, all demonstrated robust ability to activate both human and mouse Mincle-expressing HEK cells (Supplemental Figure 5). The relative activity of TDM and TDB in the human and mouse HEK cells is consistent with the results reported by others (20). Of note, the TDB and sTDCM were more potent in the mouse vs. the human Mincle receptor transfected HEK cells (Supplemental Figure 5). Interestingly, the Mincle agonist synthetic glycerol monomycolate (MMG) (26) did not display human specificity in this assay as it was able to activate both human and mouse Mincle receptor on HEK cells and was in fact more potent in mouse Mincle HEK cells (Supplemental Figure 5). This synthetic version is a shorter chain version of the lipid-derived one reported to be “human-specific” (28).

For the αTDE compound library, Mincle/NF-κB-driven SEAP reporter activity generally fell into three categories: shorter-chain compounds, B4-B14, mid-chain compounds, B16-B32, and longer chain compounds, B34-B46. Compounds B4-B14 were unable to induce measurable SEAP production from hMincle expressing HEK cells across the broad dose range evaluated (Figure 2A). These short chain compounds were weakly stimulatory with the mMincle HEK cells (Figure 2D). A similar species trend, higher activity in murine receptor-expressing cells, was discovered with the mid-chain B16-B32 compounds. Moderate, dose-dependent SEAP induction was noted in hMincle HEK cells, but more robust activity was noted from mMincle HEK cells (Figures 2B,E). Some compounds exhibited a dose-dependent decrease in SEAP activation at higher compound concentrations suggesting feedback-inhibition or toxicity at higher compound concentrations. These atypical shapes precluded curve fitting and EC50 determination for some compounds. For those effectively evaluated, lower potency (higher EC50) for both human and murine Mincle-induced SEAP induction was noted for the longer chain compounds compared to the mid-chain compounds while higher maximum SEAP production was achieved for these longer chain compounds (Figure 2G). For all compounds diminished peak SEAP levels in the human vs. the mouse cells in response to these compounds was seen (Figure 2G).

As indicated above, non-classical dose response curves were observed for several compounds with an increase in SEAP production at lower compound concentrations that drops off at higher doses. We have previously noted a similar non-classical dose response curve when evaluating TLR7/8 ligands in the HEK system (29) and we routinely see this phenotype in primary human cells with cytokine production, mostly as a result of paracrine signaling feedback loops from various cell types. To investigate this phenotype in this system, cell viability was assessed to determine if the loss of signal at the higher doses was simply as a result of cell death induced by the compound. For some of the compounds, B8, B10, B12, and TDB, there were marked decreases in viability at the top concentrations. Many others demonstrated less substantial decreases, but were still more than 5 standard deviations away from the average of the vehicle only-treated cells. These included B14, B16, B18, B20, B22, B24, and TDM (Supplemental Figure 6). However, similar to previous investigations in the TLR7/8 HEK system (29) there was not a clear correlation between viability and compound activity suggesting a mechanism other than activation-induced cell death for this recurring phenotype in these cells.

### Distinct Structure-Activity Relationships of Trehalose Diester Compounds in Human and Mouse Cells

**Figure 3:**
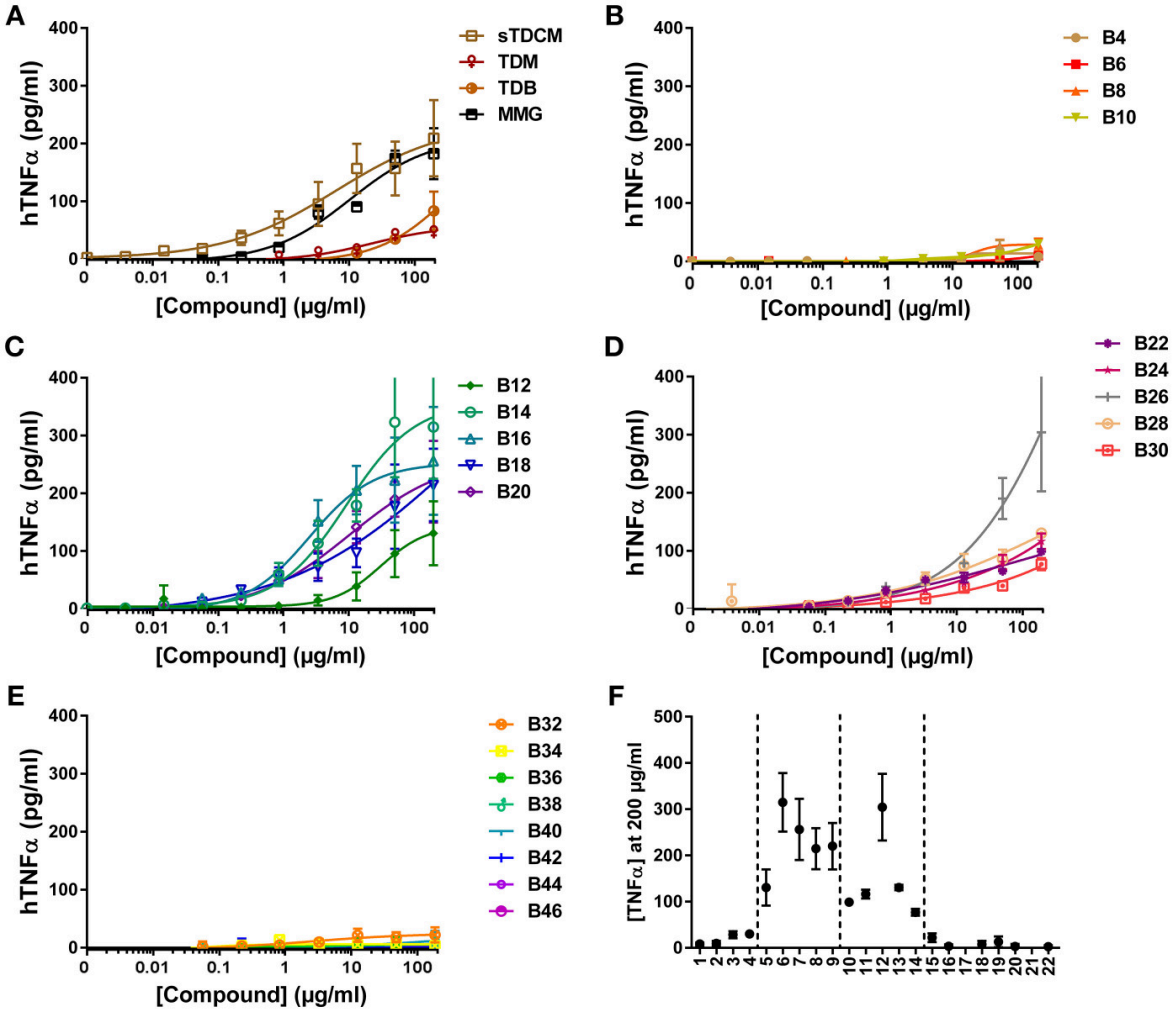
TNFα production from human THP-1 cells in response to stimulation with synthetic αTDE compounds. The indicated compounds were dissolved in 50% isopropanol/isooctane, serially diluted in vehicle and then dried to the bottom of a tissue culture plate. THP-1 cells were applied to the compound-coated plates and incubated at 37°C; supernatant was harvested 24 h later and analyzed for TNFα via ELISA **(A-E)**. Results from the 200 μg/ml concentration vs. each compounds acyl chain length are shown in **(F)**. Data points are average of at least two independent experiments ± SD.

Next, investigation into activity of the alpha-branched TDE compounds was extended to include both human and murine monocytic cell lines, THP-1 and RAW264.7, respectively. These cell lines have previously been reported as responsive to the Mincle receptor agonists TDM and TDB (30, 31). The reference compounds sTDCM and MMG demonstrated potent, robust induction of TNFα from the human THP-1 cells (Figure 3A). TDM and TDB also demonstrated activity at the highest concentrations tested, but were less potent and efficacious than the other known Mincle agonists. Biological activity of the synthetic α-branched trehalose diesters demonstrated a clear chain-length and dose-dependent activity profile. The shortest compounds, B4-B10 with chain lengths of 1–4 carbons, and longest compounds, B32-B46 with chain lengths of 15–22 carbons, were inactive for induction of TNFα from human THP-1 cells (Figures 3B,E,F). In contrast, compounds with a single chain length of 5 carbons, B12, through a chain length of 14 carbons, B30, demonstrated robust dose-responsive TNFα cytokine production. These “mid-chain length” compounds fell further into two categories with B12-B20 demonstrating the highest potencies and greatest maximum cytokine levels (Figures 3C,F). The remaining mid-chain length compounds, B22-B30, were markedly less potent, demonstrating an almost 4-fold increase in average EC50 of the groups, and less efficacious at inducing TNFα, average peak levels of the groups differing ~2-fold from 105.75 pg/ml (±21) to 228.97 pg/ml (±83) (Figures 3D,F). The only outlier to these general structure activity relationship (SAR) trends was the compound B26, which induced roughly equivalent levels of TNFα as B14/B16.

**Figure 4:**
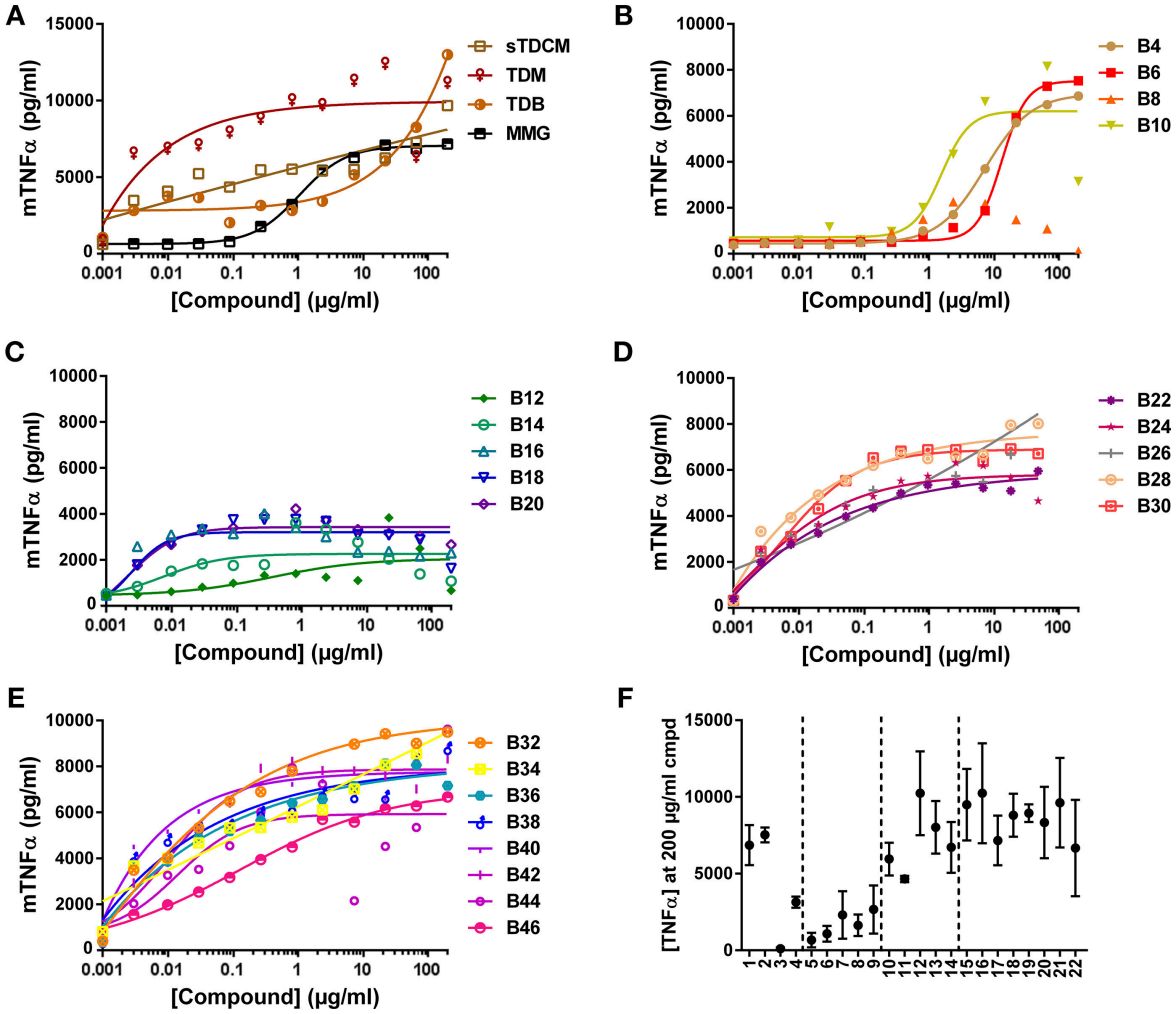
TNFα production from mouse RAW 264.7 cells in response to stimulation with synthetic αTDE compounds. The indicated compounds were dissolved in 50% isopropanol/isooctane, serially diluted in vehicle and then dried to the bottom of a tissue culture plate. RAW 264.7 cells were applied to the compound-coated plates and incubated at 37°C; supernatant was harvested 24 h later and analyzed for TNFα via ELISA **(A-E)**. Results from the 200 μg/ml concentration vs. each compounds acyl chain length are shown in **(F)**. Data points are average of at least three independent experiments, for ease of interpretation ± SEM are only displayed in **(F)**.

The compounds were also assessed in a murine macrophage cell line, RAW 264.7. Absolute TNFα values in response to the compounds from experiment to experiment varied greatly (Figure 4F), but curve shapes and compound SAR trends remained consistent between replicates. In the murine cells all control compounds, except MMG, were highly potent inducers of TNFα, demonstrating almost half maximal responses at the lowest concentrations tested (Figure 4A). The short chain length compounds, B4-B10, demonstrated some activity but with highly diminished potency compared to longer chain compounds, with an average of 15-fold increase in EC50 (Figure 4B). Similar to the human THP-1 cells the mid chain-length compounds, B12-B20, were very active (low EC50) but displayed roughly 2-fold lower maximal levels of TNFα (Figures 4C,F). In contrast to the results in human THP-1 cells, the remaining long-chain length compounds, B22-B46, were all very potent and efficacious for induction of TNFα from the murine RAW 264.7 cells (Figures 4E,D). These results are consistent with the human and murine activity previously noted in the HEK cell reporter system.

### Cytokine Profile Induced by αTDE Compounds in Cell Lines and Primary Human PBMCs

In addition to the pro-inflammatory cytokine TNFα, production of several other Th17/Th1-stimulating cytokines were evaluated in response to the TDE library in a multiplex assay from both murine and human cell lines. Interestingly, in response to the αTDE compounds THP-1 cells only produced IL-1β and TNFα and none of the other cytokines examined (IL-6, IL-23, IL-12p70, IFNγ (Supplemental Figure 7). However, the relative SAR between the compounds and cytokine production demonstrated with the TNFα ELISA was recapitulated in the multiplex assay for both TNFα and IL-1β with the mid-chain length compounds being the most active. The multiplex experiment was also performed in the murine cell line and demonstrated similar results as the human cell line where production of only TNFα was detected from the murine cell line (Supplemental Figure 8).

**Figure 5:**
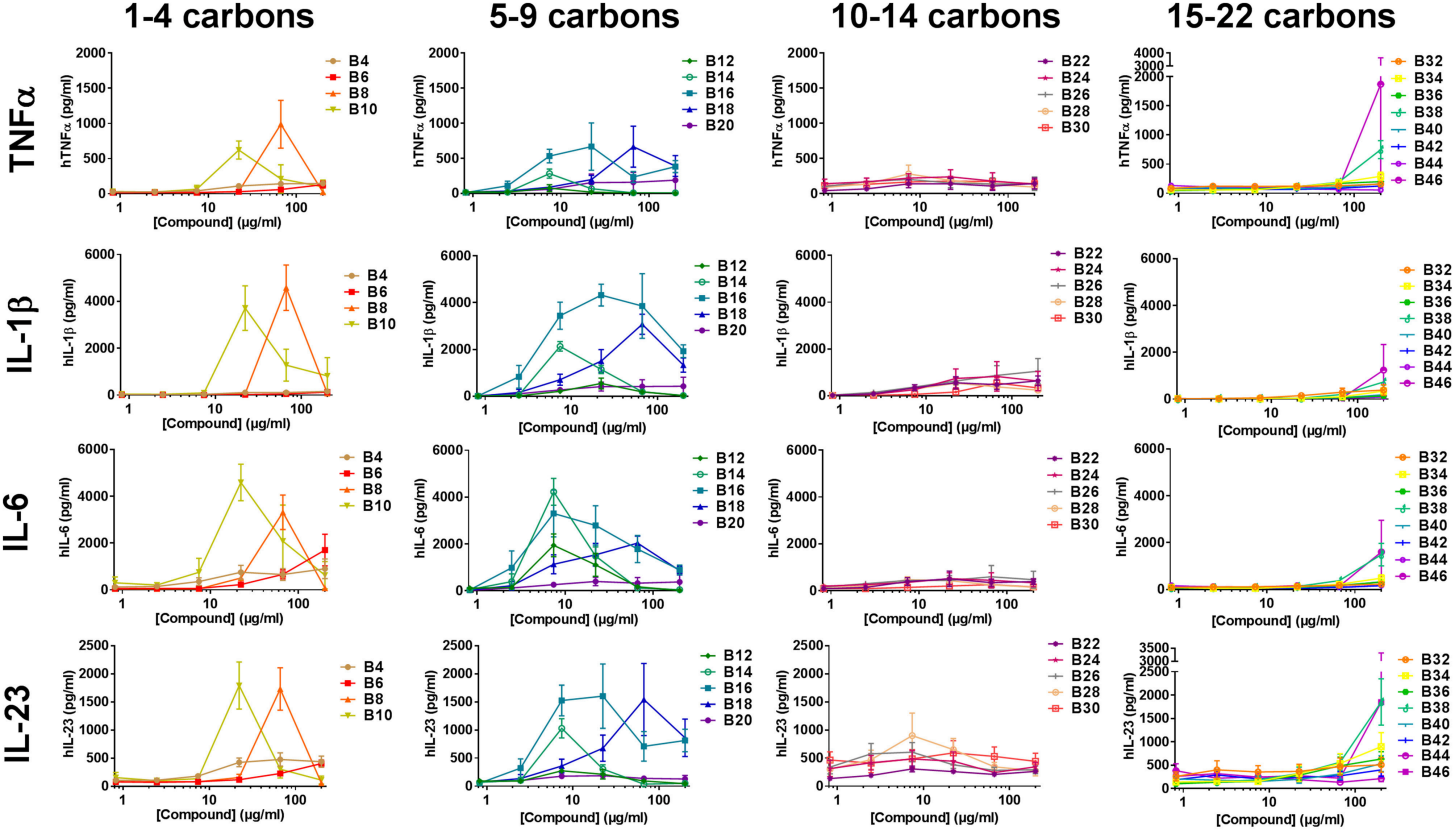
Cytokine production from primary human mononuclear cells in response to stimulation with synthetic trehalose diester compounds. The indicated compound was dissolved in 50% isopropanol/isooctane, serially diluted in vehicle and then dried to the bottom of a tissue culture plate. Cryopreserved PBMCs were thawed and applied to the compound-coated plates and incubated at 37°C; supernatant was harvested 24 h later and analyzed for TNFα, IL-1β, IL-23, IL-6. Dashed lines depict the level of cytokine of the lowest standard and thus the limit of detection of a given analyte. Results represent the mean ± SEM of 5 individual donors.

To determine if the abridged cytokine repertoire induced by the compounds from the cell lines was a cell-specific or compound-specific phenomenon, the response of a heterogeneous primary human PBMCs to the αTDE compound library was also evaluated. Primary human PBMCs were incubated with the αTDE compound and supernatants were evaluated by multiplex cytokine ELISA for TNFα, IL-1β, IL-23, IL-6 (Th17 associated) and IFNγ and IL-12p70 (Th1 associated). As with THP-1 cells, robust dose-responsive induction of TNFα and IL-1β was detected, but unlike the THP-1 cells dose-responsive stimulation of IL-6 and IL-23 production was also noted in response to a subset of the αTDE compounds (Figure 5). No production of the Th1-associated IFNγ or IL-12p70 were detected (data not shown). The relationship between compound structure and cytokine production was similar to what was previously noted in the human THP-1 cell line, though some differences were observed. While B4-B10 were largely inactive in human THP-1 cells (Figure 3B and Supplemental Figures 6A,B), Th17-associated cytokines were readily detected from human PBMCs in response to B10 and B8 (Figure 5, left graphs). The αTDE compounds B6 and B4 gave mixed responses, inactive with some donor PBMCs and low activity in others. Consistent with the previous THP-1 cell responses, the longer chain length compounds, B32-B46, induced minimal cytokine responses only at the highest dose evaluated (Figure 5, right graphs). Again as previously noted in THP-1 cells, the mid-chain length compounds B12-B20 were the most potent and efficacious at inducing all Th17 promoting cytokines (TNFα, IL-1β, IL-6, and IL-23) across a wide dose range of compound (Figure 5, 2nd column from left).

### αTDE B16 Induces Significant Th17 Immunity to *M. tuberculosis* Antigen M72

The pattern of cytokine production noted above from the human PBMCs suggests lead αTDE compounds could be effective vaccine adjuvants to induce a Th17 adaptive immune response. Based on these promising *in vitro* results, the lead candidate αTDE compound B16 (with high human potency) and B42 (with high mouse potency) were evaluated for adjuvant activity in combination with the *Mycobacteria tuberculosis* antigen, M72. M72 is a fusion protein consisting of two mycobacterial proteins Mtb32a and Mtb39a which are expressed in BCG and *M. tuberculosis* strains (32). M72 contains human CD4^+^ and CD8^+^ T cell epitopes which are highly conserved in over 45 *Mycobacteria* strains, including multi- and extreme-drug resistant strains (33). For this study, groups of 10 BALB/c mice were vaccinated intramuscularly (i.m.) two times with 14 days between the primary and booster vaccination. Increasing doses of B16 and B42 compounds were evaluated in DDA:DSPC liposomes. The known synthetic Mincle agonist TDB in a DDA:DSPC liposome was included for comparison to a *Mtb* adjuvant currently under clinical evaluation. Similar to the studies previously reported (20, 21, 24), initial *in vitro* analysis was done using plate coated compounds and subsequent *in vivo* studies were performed with liposomal formulations to optimize the structural presentation of the adjuvants and due to the physiochemical properties (lack of aqueous solubility) for this class of compounds. In contrast to previous studies with TDB liposomes (CAF01 and other related formulations) the liposomes used herein were processed using sonication (66°C) until the particle size (Zavg) was below 100 nm as measured by dynamic light scattering (Supplemental Figure 2), and were then sterile filtered by syringe 0.22 μm PVDF filtration into sealed, sterile, depyrogenated glass vial. To ensure activity of liposomal formulated compounds, liposomes formulated for *in vivo* studies were screened on murine RAW cells to assess their responsiveness. The liposome formulations were active in RAW cells (Supplemental Figure 9) but showed some differences in relative potency in comparison to the plate-coated activity. Unlike the plate-coated responses (Figure 4), B16 formulated in a DDA:DSPC liposome induced higher levels of TNFα secretion compared to B42. This result is not surprising since Mincle agonist activity is highly dependent on formulation.

**Figure 6:**
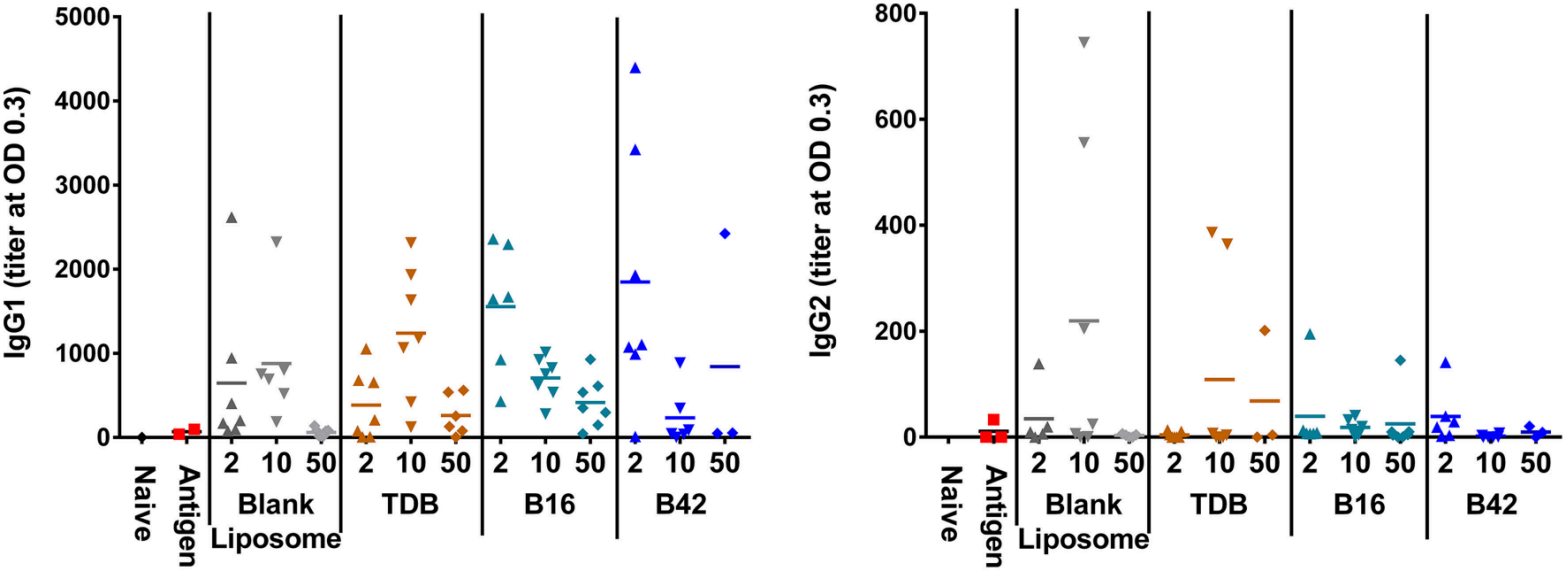
Serum antibody levels from mice in response to vaccination with recombinant M72 antigen and various αTDE compounds. BALB/c mice, 10 per group, were vaccinated two times i.m. with 0.125 μg antigen plus 2, 10, or 50 nmol of the indicated adjuvant. Serum was analyzed from 7 mice at 14 days post-secondary vaccination for presence of anti-M72 IgG1 and IgG2a antibodies via ELISA.

Anti-M72 ELISAs were performed on serum from blood collections at 14 days post-secondary vaccination to assess the antibody response to M72 antigen without and with various liposomally formulated αTDE compounds. Results from the ELISAs demonstrated modest levels of anti-M72 serum IgG1 and IgG2a antibody (Figure 6). Antigen alone induced negligible antibody titers and the addition of adjuvants boosted the response, though not significantly higher than the liposome vehicle controls. However, higher levels of IgG1 antibodies were seen with all αTDE compounds suggesting a potential Th2 biased immune response.

**Figure 7:**
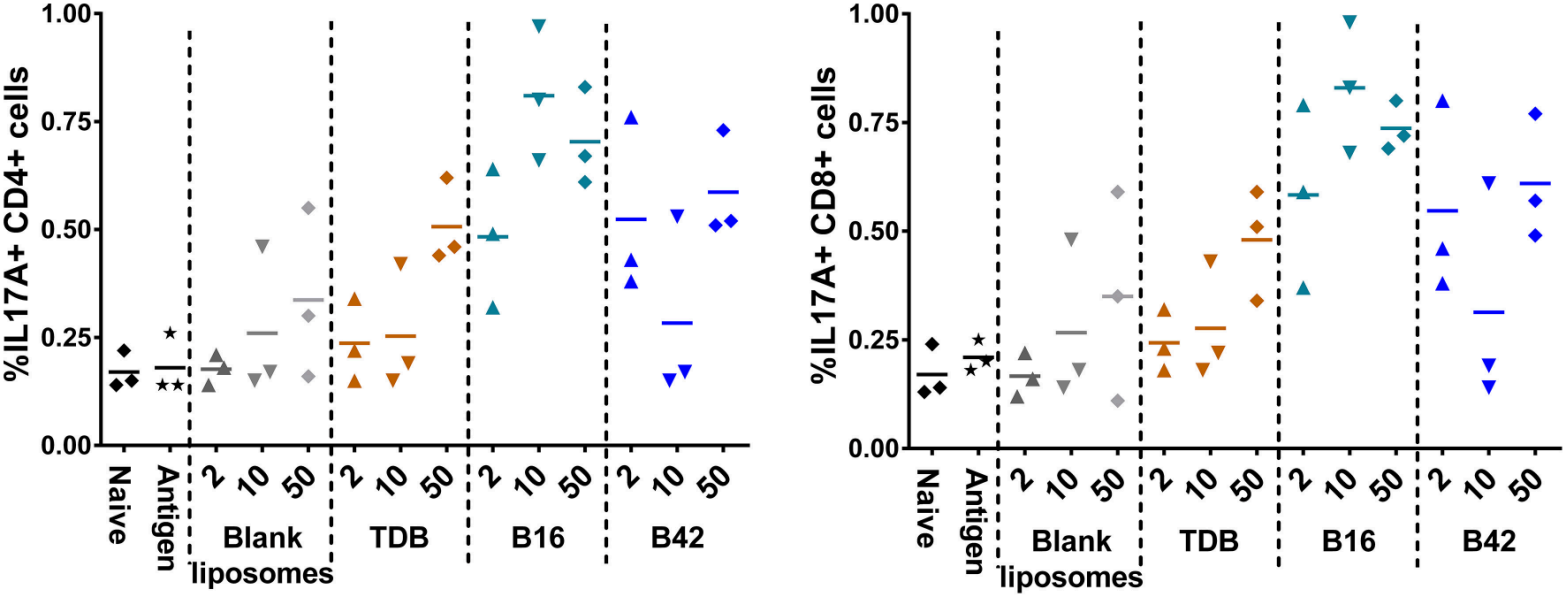
Induction of IL-17 producing CD4+ and CD8+ T cells from an αTDE adjuvanted M72 vaccine. BALB/c mice, 10 per group, were immunized two times i.m. with 0.125 μg antigen plus 2, 10, or 50 nmol of the indicated adjuvant. Spleens were harvested from a subset of 3 mice per group at 5 days post-secondary vaccination. Splenocytes were restimulated with 1 μg/mL whole antigen and transport inhibitors followed by surface staining for CD3, CD4, and CD8 and then intracellular cytokine staining. Data represent percentage of live, CD3^+^/CD4^+^, left, or CD3^+^/CD8^+^, right, cells that are also positive for IL-17A upon stimulation.

To assess the M72-specific CD4^+^ and CD8^+^ T cell responses generated in response to the adjuvants, spleens were harvested from a subset of mice 5 days post-secondary vaccination and re-stimulated with whole M72 antigen and protein transport inhibitors followed by intracellular cytokine staining. Effector antigen-specific T cells were defined by their cytokine induction profile upon recall: Th1 T cells were identified as IFNγ producers supported by the production of IL-2 and TNFα, Th2 cells were defined as IL-5 producers and Th17 cells as IL-17A producers. Mice vaccinated with B16 and M72 antigen exhibited the greatest frequency of IL-17+ cells in both CD4^+^ and CD8^+^ T cells following antigen re-stimulation (Figure 7). Frequencies of IFNγ producing T cells were not increased in comparison to naïve mice in any of the vaccinated groups (Supplemental Figure 10), suggesting that a Th1 response is not induced by adjuvant plus M72 combinations. IL-5 production was increased in CD8^+^ T cells with 50 nmol B42 (over naïve and liposome control) and 2 nmol B42 (over liposome control; Supplemental Figure 10) suggesting a Th2 skewing with B42. TNFα production was increased compared to controls in the B42 adjuvanted groups at the highest dose evaluated while IL-2 levels were moderately increased over antigen alone in most adjuvanted groups (Supplemental Figure 10). The frequency of multifunctional (IFNγ^+^ IL-2^+^ TNFα^+^) CD4+ and CD8+ T cells was also assessed. The frequency of these cells was very low and few changes were noted (data not shown).

**Figure 8:**
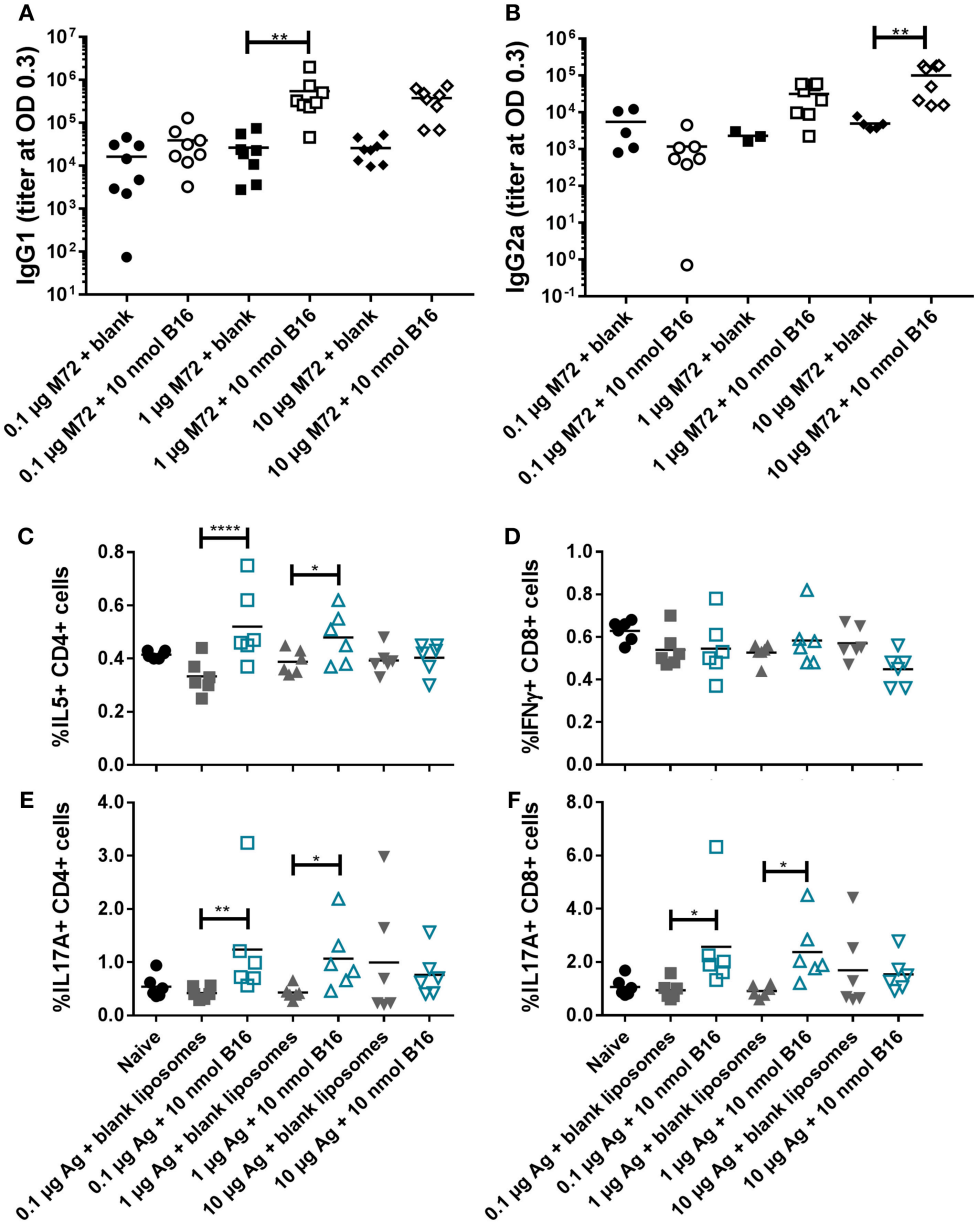
B16 liposomes biases a Th17/Th2 adaptive immune response. BALB/c mice, 14 per group, were immunized twice, 14 days apart, i.m with 0.1, 1, or 10 μg of M72 antigen plus either blank DDA:DSPC liposome or B16 loaded (10 nmol final dose) liposome. **(A,B)** M72-specific serum antibody titers at 14 days post-secondary vaccination. **(C–F)** Spleens were harvested from mice at 5 days post-secondary vaccination and restimulated with 1 μg/mL whole antigen and transport inhibitors followed by surface staining for CD3, CD4, and CD8 and then intracellular cytokine staining. **(C)** Percent of live, CD3^+^/CD4^+^/IL5+ cells, **(D)** Percent of live, CD3^+^/CD8^+^/IFNγ+ cells, **(E)** Percent of live, CD3^+^/CD4^+^/IL17+ cells, and **(F)** Percent of live, CD3^+^/CD8^+^/IL17+ cells. ^*^p < 0.05, ^**^p < 0.01, ^****^p < 0.001.

Based on the promising *in vivo* activity of B16, a comprehensive *in vivo* study examining the adjuvant activity of B16 liposomes with varying doses of M72 antigen was completed. The low M72-specific antibody titers observed in the initial *in vivo* study (Figure 6) as well as increased but variable frequencies of M72-specific IL-17+ T cells led us to design a study in which the antigen dose was increased while the dose of B16 was held at 10 nmol based on the previous study findings. M72 specific serum IgG1 and IgG2a antibody titers (measured at 14 dp2) were significantly improved with increasing doses of M72 antigen in the presence of B16 adjuvant (Figures 8A,B). Similar to the previous study, the lowest dose of M72 antigen (0.1 μg) did not exhibit significantly higher antibody titers in the presence of B16 adjuvant, although significantly higher antigen-specific T-cell responses were observed (see below). These data clearly demonstrate a dose-dependent increase in anti-M72 serum antibody titers in the presence of B16 liposome adjuvant.

M72 restimulation of splenocytes confirmed results from the first *in vivo* study demonstrating liposomal B16 induces significantly higher frequencies of IL-17+ CD4 and CD8 T cells with 0.1 μg M72/mouse and 1 μg M72/mouse compared to antigen plus blank liposomes (Figures 8E,F). At the highest antigen dose (10 μg M72), these differences were not significant due to higher responses from the antigen plus blank liposome (Figures 8E,F). Frequencies of IL-5+ CD4 T cells are similarly increased in the two lower doses of antigen plus B16 compared to antigen plus blank liposome (Figure 8C), but again no difference is observed at the highest antigen dose tested (Figure 8C). Frequencies of IL-5+, IL-2+, and TNFα+ CD8 T cells are also increased in the lower doses of antigen plus B16 compared to antigen plus blank liposome, but not at the highest dose (Supplemental Figure 11B). IL-2+ CD4 T cells were similarly increased only in the middle dose of antigen plus B16 compared to blank antigen while frequencies of TNFα+ CD4 T cells were not increased in any of the B16 groups compared to their antigen matched blank liposome groups (Supplemental Figure 11A). Similar to the previous study, no differences in frequencies of IFNγ+ CD8 T cells (Figure 8C) or IFNγ+ CD4 T cells (Supplemental Figure 11A, top) were observed between groups. Collectively, these *in vivo* studies show the Th2 and Th17 adjuvanting capacity of liposomal αTDE B16.

## Discussion

In this study we examined the effect that acyl chain length has on biological activity of alpha-branched trehalose diester compounds in both human and mice. A library of molecules was evaluated for their ability to bind and activate the C type lectin receptor Mincle, induce cytokine production from mouse and human macrophage cell lines, and primary human PBMCs. The most active compounds induced a Th17-skewing cytokine pattern from primary human cells suggesting that they could serve as Th17-promoting adjuvant molecules. This finding was supported through *in vivo* murine studies using a clinical candidate *Mtb* antigen.

Several studies of trehalose glycolipids have been performed to date to assess the structure activity relationship between this class of compounds and the immunostimulatory activity they exert (22–25). These studies have been plagued by several limitations including incomplete compound libraries for comprehensive analysis of structure activity relationships within a single series (i.e., alpha branched diesters). In addition, complete biological evaluation of the compounds in both human and mouse cells has been lacking. In previously published *in vivo* studies, Mincle agonists tend to bias a Th1/Th17 response which is in contrast to our findings of a Th2/Th17 response. The loss of Mincle has been shown to reduce IFNγ in an infection setting (34) and when DDA liposomes are used as a delivery system, TDB and other Mincle ligands have been shown to bias a Th1/Th17 vaccine immune response (16, 24, 35). While these groups used C57BL/6 mice, a Th1-biased mouse strain, Balb/c mice, a Th2-biased mouse strain, were used for the studies reported herein. Furthermore, route of administration (s.c. vs. i.m.) and antigen used could account for differences in results between this study and those previously reported. Differences could also arise from the specific liposome formulation and processing to a size range amenable to sterile filtration. In our experience DDA liposomes are unstable, non-uniform, and difficult to reproduce so the liposomal formulations developed for these studies include DSPC to stabilize the liposome and allow for stable particles below 100 nm which can be readily sterilized using a 0.22 μm PVDF filter. However, DDA liposomes have been shown to efficiently adsorb antigen (36) and whether the addition of DSPC reduces that adsorption with our formulations is not know. Further, co-delivery of antigen and adjuvant is important for biasing a Th1 response (37). It is therefore possible that adsorption of antigen by liposomal formulations in this study was not efficient, which may have led to a lack of antigen and adjuvant co-delivery, reducing the Th1 bias of the immune response. Further, DDA liposomes are much larger (36) than liposomes used here, which may lead to differences in trafficking and subsequent immune response.

Several seminal studies on trehalose glycolipids have investigated the function of these compounds. Trehalose monoesters are able to activate macrophages in a Mincle-dependent fashion (23) but the diester forms are superior in their immunostimulatory capacity (24). While these studies elegantly demonstrate the utility of the number of acyl chains decorating the trehalose, they fail to exhaustively address the effect of chain length on activity; furthermore they perform all their analyses in murine cells. Khan et al. completed a detailed analysis of un-branched chain lengths from 2 to 20 carbons in murine cells (22). These studies are consistent with the findings reported here in that the longer-chain lipids are more active at cytokine induction from mouse cells suggesting that the branching is less important for activity in murine cells than acyl chain length (Figure 4) (22, 38).

Most of the prior Mincle agonist SAR studies focus exclusively on murine cells. As reported herein, SAR findings for the varying chain length αTDE compounds was strikingly different between the murine and human cells. In the murine cells the longer chain compounds displayed the highest immunostimulatory activity, the mid-chain length compounds were more potent (lower EC50) but appeared to function as partial agonists as demonstrated by a decreased maximum cytokine output. In the human cells, the longer chain molecules were largely inactive and the greatest activity came from a small window of 5–14 length carbon chains, compounds B12-B30. The human results were recapitulated in both the macrophage cell line and the heterogenous primary cell population (Figures 3,5). The same species distinction was not seen in the human vs. murine Mincle-expressing HEK cells (Figure 2); these differences suggest the transfected Mincle receptors or chimeric mouse receptor with human signaling components do not reflect the same receptor binding or signaling as murine and human cells. Alternatively, these compounds could be signaling through another C-Type Lectin Receptor in addition to Mincle receptor in primary human cells and cell lines. Additional studies are needed to further explore these possibilities. The trends for cell activation in response to the various compounds was very similar between the two species in this HEK system. This distinction is likely a result of other co-receptors or signaling components that are present on macrophages and other immune cell types but not in the transgenic HEK system. It is widely known that Mincle agonists work through and with other innate immune receptors, such as MCL, to exert their effect and these receptors could be the drivers behind the structure activity differences seen between species, rather than direct Mincle interaction (34). These results underscore the importance of analysis of structurally variant compounds not only in disparate species, but also in the cell type(s) that will most likely be the target of the compound.

The identification and development of novel synthetic adjuvants capable of inducing a Th17 or Th1/Th17 adaptive immunity with a co-delivered antigen will provide new opportunities for the development of vaccine candidates targeting bacterial and/or fungal infections. Several diseases of significant medical concern could benefit from a Th17 inducing adjuvant system including *Mycobacterium tuberculosis* (Mtb), *Staphylococcus aureus, Pseudomonas aeruginosa, Streptococcus pneumonia, Candida albicans, Aspergillus fumigatus* and others. To this end, we evaluated the lead candidate alpha-branched trehalose diester compounds B16 and B42 with a clinical candidate Mtb fusion antigen, M72. Surprisingly, neither compound significantly enhanced serum antibody titers when a low dose of antigen (0.125 μg) was used suggesting Mincle based adjuvant systems alone may not provide much of a dose sparing effect for humoral immunity. Significantly higher serum antibody titers were noted when higher antigen doses were evaluated (Figure 8). However, induction of antibody responses in *Mtb* vaccines has not been found to be protective in a vaccine or natural infection setting, whereas Th17 or Th1/Th17 immunity has been reported to play a strong role in protection from *Mtb* (39). As expected based on the *in vitro* cytokine activity from the macrophage cell lines, both compounds were able to drive superior Th17 responses, though B16 was more effective (Figure 7). In contrast to the antibody data indicating that increasing antigen leads to increased antibody responses, we did not find this to be true for T cell responses (Figures 8C–E) where the strongest Th17 responses were noted at lower antigen doses. These results were also compared with mice vaccinated using the clinical candidate Mincle agonist TDB. TDB was also able to boost Th17 in mice as previously reported (35), but did so with diminished potency over the αTDE compounds. This data supports the *in vitro* results and suggests that optimization of structure can enhance potency and potentially vaccine efficacy. Collectively, this study demonstrates the utility of mid-chain length αTDE compounds as Th17-inducing vaccine adjuvants for *Mtb* vaccines.

## Ethics Statement

All animal studies were carried out in accordance with the recommendations and approval of University of Montana's Institutional Animal care and Use Committee (IACUC). Blood from healthy human subjects was obtained with informed consent in accordance with the recommendations and approval of University of Montana's Institutional Review Board (IRB).

## Author Contributions

JE, CB, RC, SM, MW, and AS designed experiments and analyzed data. GE and KR synthesized TDE compounds and assisted with structure-activity relationship data analysis. RS and DB provided formulation and analytical chemistry expertise. AS and JE wrote the manuscript draft. All authors discussed data, reviewed and edited the final manuscript.

### Conflict of Interest Statement

JE, CB, RC, GE, RS, MW, DB, KR, and AS were, at the initiation of the studies outlined herein, employees of the GlaxoSmithKline Vaccines. JE, GE, DB, KR, and AS contributed to or are listed as inventors on patents owned by GSK in connection with the compounds reported in this manuscript. The remaining author declares that the research was conducted in the absence of any commercial or financial relationships that could be construed as a potential conflict of interest.
