# Supplementary material for: Species-Specific Structural Requirements of Alpha-Branched Trehalose Diester Mincle Agonists
Source: Front Immunol. 2019 Feb 28;10:338. doi: 10.3389/fimmu.2019.00338 (PMC6403188; doi:10.3389/fimmu.2019.00338)
Supplement: Supplementary file 1 [file Data_Sheet_1.docx]

B4 pyridine-d5 1H NMR (400 MHz): 5.87 (d, *J*= 3.7 Hz, 2H), 5.07 (dd, *J*= 10.1, 4.4 Hz, 2H), 5.00 (d, *J*= 11.3 Hz, 2H), 4.79 (dd, *J*=11.7, 5.2 Hz, 2H), 4.73 (t, *J*= 9.3 Hz, 2H), 4.28 (dd, *J*= 9.3, 3.2 Hz, 2H), 4.13 (t, *J*= 9.3 Hz, 2H), 2.54 (m, 2H), 1.11 (t, *J*= 7.1 Hz, 12H)

B6 pyridine-d5 1H NMR (400 MHz): 5.85 (b, 2H), 5.04 (d, *J*=11.2 Hz, 4H), 4.82 (dd, *J*= 11.5, 4.5 Hz, 2H), 4.74 (t, *J*=8.9 Hz, 2H), 4.27 (d, *J*= 8.7 Hz, 2H), 4.16 (t, *J*=8.9 Hz, 2H), 2.27 (m, 2H), 1.66 (m, 4H), 1.46 (m, 4H), 0.87 (t, *J*= 6.9 Hz, 12H)

B8 pyridine-d5 1H NMR (400 MHz): 5.85 (d, *J*= 3.4 Hz, 2H), 5.06 (d, *J*= 10.4 Hz, 4H), 4.82 (dd, *J*= 11.8, 5.3 Hz, 2H), 4.73 (t, *J*= 9.0 Hz, 2H), 4.27 (dd, *J* = 9.4, 3.4 Hz, 2H), 4.15 (t, *J*= 9.2 Hz, 2H), 2.49 (m, 2H), 1.68 (m, 4H), 1.36 (b, 12H), 0.82 (t, *J*= 6.9 Hz, 12H)

B10 pyridine-d5 1H NMR (400 MHz): 5.86 (b, 2H), 5.07 (d, *J*= 10.4 Hz, 4H), 4.85 (dd, *J*=11.4 4.1 Hz, 2H), 4.72 (t, *J*= 9.1 Hz, 2H), 4.28 (d, *J*= 9.7 Hz, 2H), 4.18 (t, *J*= 9.2 Hz, 2H), 2.48 (m, 2H), 1.72 (b, 4H), 1.46 (b, 4H), 1.28 (b, 16H), 0.82 (b, 12H)

B12 pyridine-d5 1H NMR (400 MHz): 5.85 (b, 2H), 5.04 (d, *J*=12.5 Hz, 4H), 4.95 (b, 2H), 4.85 (d, *J*= 9.6 Hz, 2H), 4.74 (t, *J*= 8.5 Hz, 2H), 4.28 (d, *J*= 8.5 Hz, 2H), 4.18 (t, *J*=7.9 Hz, 2H), 2.50 (m, 2H), 1.74 (b, 4H), 1.49 (b, 4H), 1.37 (b, 8H), 1.22 (b, 16H), 0.83 (b, 12H)

B14 pyridine-d5 1H NMR (400 MHz): 5.86 (d, *J*=3.5 Hz, 2H), 5.05 (d, *J*= 11.0 Hz, 4H), 4.87 (dd, *J*= 11.9, 4.8 Hz, 2H), 4.74 (t, *J*= 9.1 Hz, 2H), 4.28 (b, 2H), 4.19 (m, 2H), 2.54 (m, 2H), 1.77 (b, 4H), 1.53 (b, 4H), 1.38 (b, 8H), 1.22 (b, 24H), 0.85 (t, *J*= 6.5 Hz, 12H)

B16 pyridine-d5 1H NMR (400 MHz): 5.87 (d, *J*= 3.1 Hz, 2H), 5.07 (d, *J*= 11.5 Hz, 4H), 4.88 (dd, *J*= 12.2, 5.1 Hz, 2H), 4.75 (t, *J*= 9.2 Hz, 2H), 4.30 (d, *J*= 8.9 Hz, 2H), 4.20 (t, *J*= 8.9 Hz, 2H), 1.81 (b, 4H), 1.55 (b, 4H), 1.42 (b, 8H), 1.25 (b, 32H), 0.88 (t, *J*= 6.9 Hz, 12H)

B18 pyridine-d5 1H NMR (400 MHz): 5.87 (b, 2H), 5.06 (d, *J*= 10.2 Hz, 4H), 4.88 (d, *J*= 11.0, 2H), 4.74 (t, *J*= 8.9 Hz, 2H), 4.29 (d, *J*= 9.5 Hz, 2H), 4.19 (t, *J*= 8.0 Hz, 2H), 2.57 (m, 2H), 1.80 (b, 4H), 1.56 (b, 4H), 1.42 (b, 8H), 1.23 (b, 40H), 0.88 (b, 12H)

B20 pyridine-d5 1H NMR (400 MHz): 5.88 (b, 2H), 5.08 (b, 4H), 4.88 (b, 2H), 4.74 (b, 2H), 4.30 (b, 2H), 4.20 (b, 2H), 2.59 (b, 2H), 1.82 (b, 4H), 1.57 (b, 4H), 1.44 (b, 8H), 1.25 (b, 48H), 0.89 (b, 12H)

B22 pyridine-d5 1H NMR (400 MHz): 5.88 (d, *J*= 3.4 Hz, 2H), 5.08 (d, *J*= 10.3 Hz, 4H), 4.89 (dd, *J*= 11.7, 4.8 Hz, 2H), 4.74 (t, *J*= 8.8 Hz, 2H), 4.30 (dd, *J*= 9.4, 3.4 Hz, 2H), 4.20 (t, *J*= 9.4 Hz, 2H), 2.59 (m, 2H), 1.83 (m, 4H), 1.58 (m, 4H), 1.44 (b, 8H), 1.26 (b, 56H), 0.89 (t, *J*= 6.9 Hz, 12H)

B24 pyridine-d5 1H NMR (400 MHz): 5.84 (d, *J*= 3.0 Hz, 2H), 5.07 (d, *J*= 11.0 Hz, 4H), 4.88 (dd, *J*= 11.6, 4.3 Hz, 2H), 4.73 (t, *J*=8.6 Hz, 2H), 4.31 (b, 2H), 4.19 (t, *J*= 7.6 Hz, 2H), 2.58 (m, 2H), 1.83 (m, 4H), 1.58 (b, 4H), 1.45 (b, 8H), 1.26 (b, 66H), 0.88 (t, *J*= 5.5 Hz, 12H)

B26 pyridine-d5 1H NMR (400 MHz): 5.86 (d, *J*=3.6 Hz, 2H), 5.07 (d, *J*=10.8 Hz, 4H), 4.88 (dd, *J*=4.3, 11.7 Hz, 2H), 4.73 (t, *J*= 8.7 Hz, 2H), 4.29 (dd, *J*= 7.4, 4.9Hz, 2H), 4.18 (t, *J*=9.4 Hz, 2H), 2.59 (m, 2H), 2.28 (b, 2H), 1.83 (b, 4H), 1.58 (b, 4H), 1.44 (b,8H), 1.27(b, 68H), 0.89 (t, *J*=4.9 Hz, 12H)

B28 pyridine-d5 1H NMR (400 MHz): 5.86 (d, *J*= 3.2 Hz, 2H), 5.06 (d, *J*=11.3 Hz, 4H), 4.86 (dd, *J*= 11.7, 4.8 Hz, 2H), 4.72 (t, *J*= 9.2 Hz, 2H), 4.28 (dd, *J*= 9.5, 3.2 Hz, 2H), 4.17 (t, *J*= 9.4 Hz, 2H), 2.57 (m, 2H), 1.80 (m, 4H), 1.57 (m, 4H), 1.43 (m, 8H), 1.27 (b, 76H), 0.87 (t, *J*= 6.7 Hz, 12H)

B30 pyridine-d5 1H NMR (400 MHz): 5.89 (b, 2H), 5.08 (d, *J*= 10.6 Hz, 4H), 4.89 (dd, *J*= 10.4, 3.4 Hz, 2H), 4.74 (t, *J*= 8.9 Hz, 2H), 4.29 (d, *J*= 9.6 Hz, 2H), 4.19 (t, *J*= 9.4 Hz, 2H), 2.60 (m, 2H), 1.83 (b, 4H), 1.59 (b, 4H), 1.45 (b, 8H), 1.30 (b, 84H), 0.88 (t, *J*= 5.8 Hz, 12H)

B32 pyridine-d5 1H NMR (400 MHz): 5.88 (d, *J*= 3.3 Hz, 2H), 5.08 (d, *J*= 11.3 Hz, 4H), 4.88 (dd, *J*= 11.7, 4.9 Hz, 2H), 4.72 (t, *J*= 8.9 Hz, 2H), 4.29 (d, *J*= 8.9 Hz, 2H), 4.19 (t, *J*= 8.3 Hz, 2H), 2.60 (m, 2H), 1.84 (m, 4H), 1.59 (m, 4H), 1.46 (b, 8H), 1.31 (b, 92H), 0.89 (t, *J*= 7.0 Hz, 12H)

B34 pyridine-d5 1H NMR (400 MHz): 5.88 (b, 2H), 5.07 (d, *J*=10.6 Hz, 4H), 4.87 (b, 2H), 4.73 (t, *J*= 8.3 Hz, 2H), 4.30 (d, *J*= 9.5 Hz, 2H), 4.19 (t, *J*= 9.5 Hz, 2H), 2.60 (m, 2H), 1.84 (m, 4H), 1.60 (m, 4H), 1.46 (b, 8H), 1.32(b, 100H), 0.89 (b, 12H)

B36 pyridine-d5 1H NMR (400 MHz): 5.89 (b, 2H), 5.08 (d, *J*= 8.9 Hz, 4H), 4.88 (b, 2H), 4.74 (b, 2H), 4.30 (b, 2H), 4.19 (b, 2H), 2.60 (b, 2H), 1.84 (b, 4H), 1.60 (b, 4H), 1.46 (b, 8H), 1.32 (b, 108H), 0.89 (b, 12H)

B38 pyridine-d5 1H NMR (400 MHz): 5.88 (d, *J*= 3.6 Hz, 2H), 5.08 (d, *J*- 11.3 Hz, 4H), 4.88 (dd, *J*= 11.6, 5.0 Hz, 2H), 4.74 (t, *J*= 9.4, 2H), 4.30 (dd, *J*= 9.5, 3.6 Hz, 2H), 4.19 ( t, *J*= 9.2, 2H), 2.60 (m, 2H), 1.85 (m, 4H), 1.59 (m, 4H), 1.47(b, 8H), 1.29 (b, 116H), 0.89 (t, *J*= 6.9 Hz, 12H)

B40 pyridine-d5 1H NMR (400 MHz): 5.88 (d, *J*= 2.5 Hz, 2H), 5.07 (d, *J*= 10.9 Hz, 4H), 4.89 (dd, *J*= 11.5, 4.6 Hz, 2H), 4.73 (t, *J*= 8.9 Hz, 2H), 4.29 (d, *J*= 8.6 Hz, 2H), 4.16 (t, *J*= 9.1 Hz, 2H), 2.60 (m, 2H), 1.85 (m, 4H), 1.60 (m, 4H), 1.46 (b, 8H), 1.32 (b, 124H), 0.88 (t, *J*- 6.7 Hz, 12H)

B42 pyridine-d5 1H NMR (400 MHz): 5.88 (d, *J*= 3.7 Hz, 2H), 5.08 (d, *J*= 10.7 Hz, 4H), 4.88 (dd, *J*= 11.7, 5.1 Hz, 2H), 4.73 (t, *J*= 9.2 Hz, 2H), 4.30 (dd, *J*= 9.6, 3.6 Hz, 2H), 4.19 (t, *J*= 9.5 Hz, 2H), 2.60 (m, 2H), 1.84 (m, 4H), 1.59 (m, 4H), 1.46 (b, 8H), 1.32 (132H), 0.89 (t, *J*= 7.3 Hz, 12H)

B44 pyridine-d5 1H NMR (400 MHz): 5.89 (d, *J*= 3.5 Hz, 2H), 5.09 (d, *J*= 11.2 Hz, 4H), 4.89 (dd, *J*= 11.9, 5.1 Hz, 2H), 4.45 (t, *J*= 9.1 Hz, 2H), 4.32(d, *J*= 8.8 Hz, 2H), 4.20 (t, *J*= 8.8 Hz, 2H), 2.61 (m, 2H), 1.85 (m, 4H), 1.60 (m, 4H), 1.46 (b, 8H), 1.32 (b, 140H), 0.89 (t, *J*= 6.8 Hz, 12H)

B46 pyridine-d5 1H NMR (400 MHz):5.90 (b, 2H), 5.10 (d, *J*= 8.9 Hz, 4H), 4.90 (m, 2H), 4.75 (t, *J*= 8.3 Hz, 2H), 4.32 (m, 2H), 4.20 (t, *J*= 7.8 Hz, 2H), 2.60 (m, 2H), 1.85 (m, 4H), 1.61 (m, 4H), 1.47 (b, 8H), 1.34 (b, 148H), 0.88 (b, 12H)

**Supplemental Figure 1.** ^1^H NMR data of compounds in pyridine-d5 (Sigma-Aldrich) collected on an Agilent Technologies 400/54 NMR spectrometer. Data files were analyzed using Spinworks 4 software.


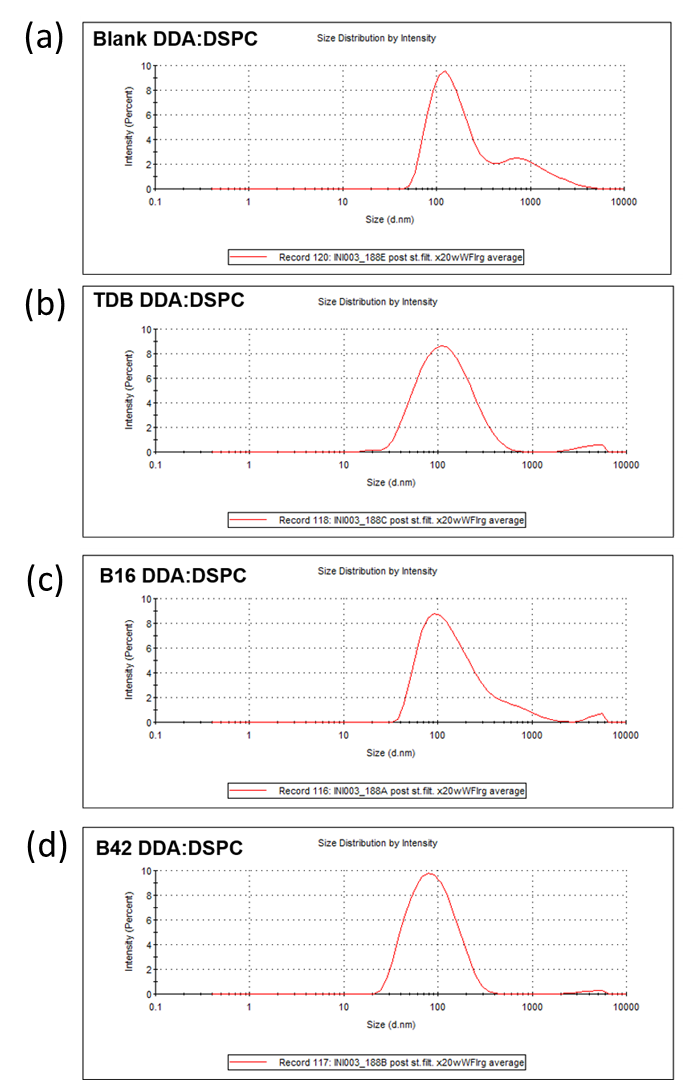


Supplemental Figure 2. Dynamic light scatter (DLS) curves for liposome characterization. Histograms of liposome size distribution determined by DLS for (a) blank DDA:DSPC, (b) TDB DDA:DSPC, (c) B16 DDA:DSPC, or (d) B42 DDA:DSPC.


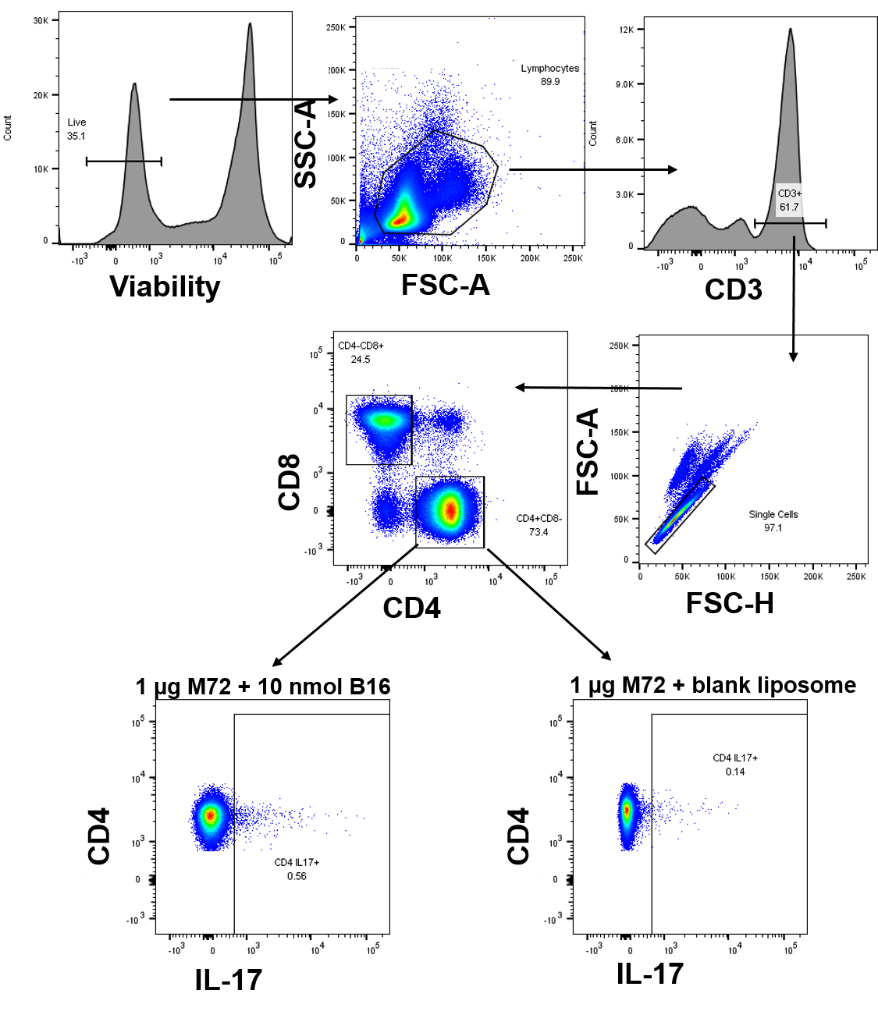


Supplemental Figure 3. Example flow gating for murine splenocyte restimulation. For *in vivo* experiments, mice were injected intramuscularly with antigen plus formulated adjuvant twice with 2 weeks between the first and second injection. 5 days after the second injection, spleens were harvested and single cell suspensions prepared. Flow cytometry was performed on restimulated splenocytes with gating as shown above. Also shown are examples of IL-17 intracellular cytokine staining comparing an adjuvanted response (1ug M72 + 10 nmol B16) to a non-adjuvanted response (1 ug M72 + blank liposome).

Supplemental Figure 4. αTDEs do not induce a response in HEK null cells. The indicated compounds were dissolved in 50% isopropanol/isooctane, serially diluted in vehicle and then dried to the bottom of a tissue culture plate. HEK null cells (not transfected with human or mouse Mincle) with an NF-κB-driven SEAP reporter were incubated with the compounds for 24 h followed by assessment of the supernatants for SEAP levels. Data are represented as fold change in OD650 over vehicle treated cells. Graphs are mean values from three independent experiments ± SEM.


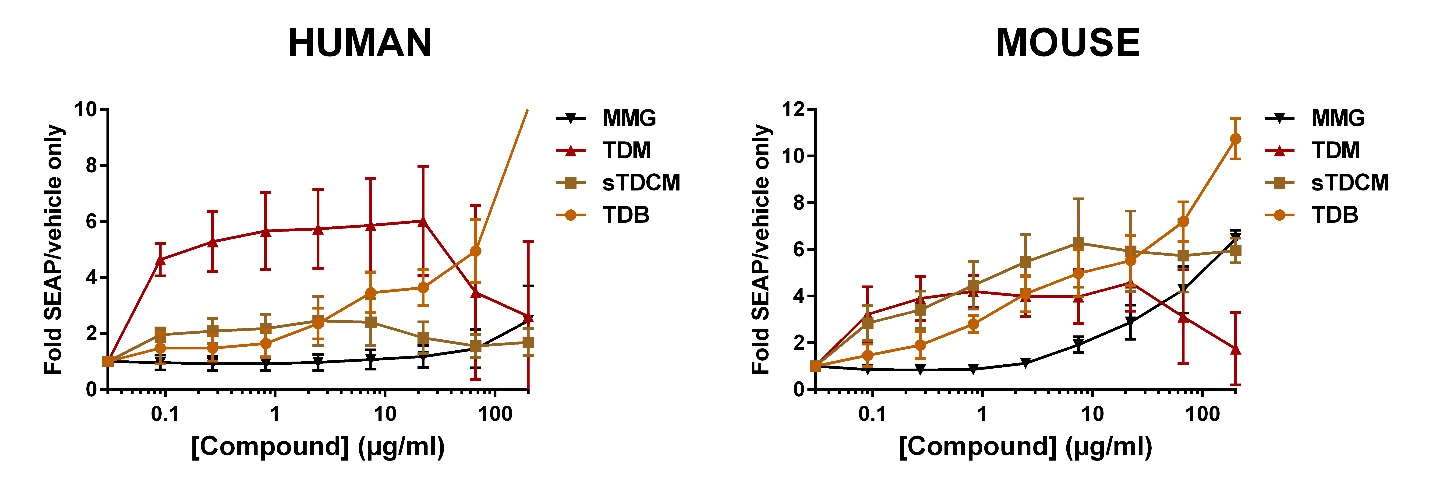


Supplemental Figure 5. Activation of human or mouse Mincle in response TDM. The indicated compounds were dissolved in 50% isopropanol/isooctane, serially diluted in vehicle and then dried to the bottom of a tissue culture plate. HEK cells transfected with human or mouse Mincle and an NF-κB-driven SEAP reporter were incubated with the compounds for 24 h followed by assessment of the supernatants for SEAP levels. Data are represented as fold change in OD650 over vehicle treated cells. Graphs are mean values from three independent experiments ± SEM.


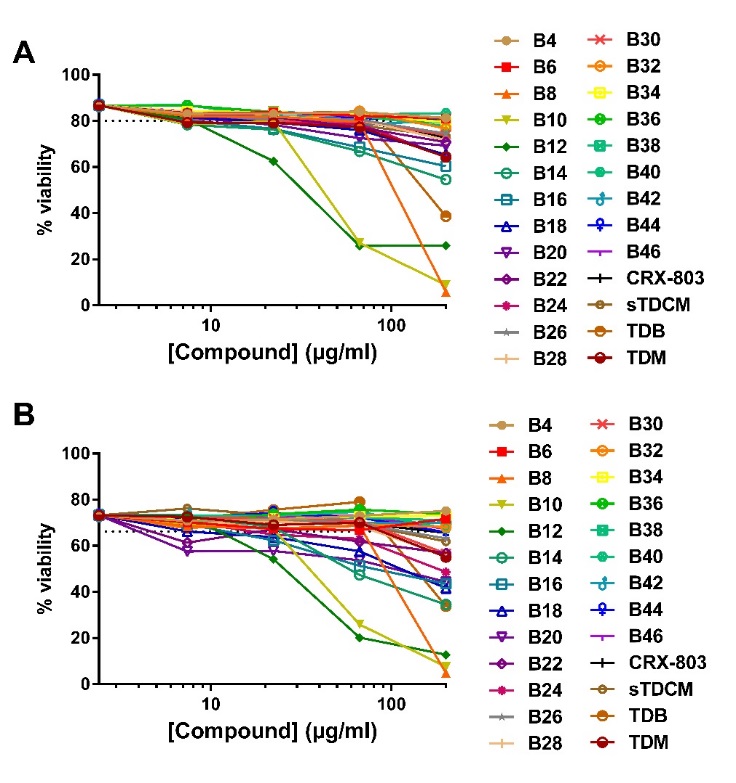


Supplemental Figure 6. Viability of Hek human and mouse Mincle-expressing cells in response to various αTDE compounds. Hek Blue human (A) or mouse (B) Mincle-expressing cells were incubated with increasing concentrations of the indicated plate coated compound for 24 h followed by assessment of viability using Live/Dead staining and analysis via FACS. Dashed lines represent values < 5x S.D. of the mean of vehicle-only treated cells.


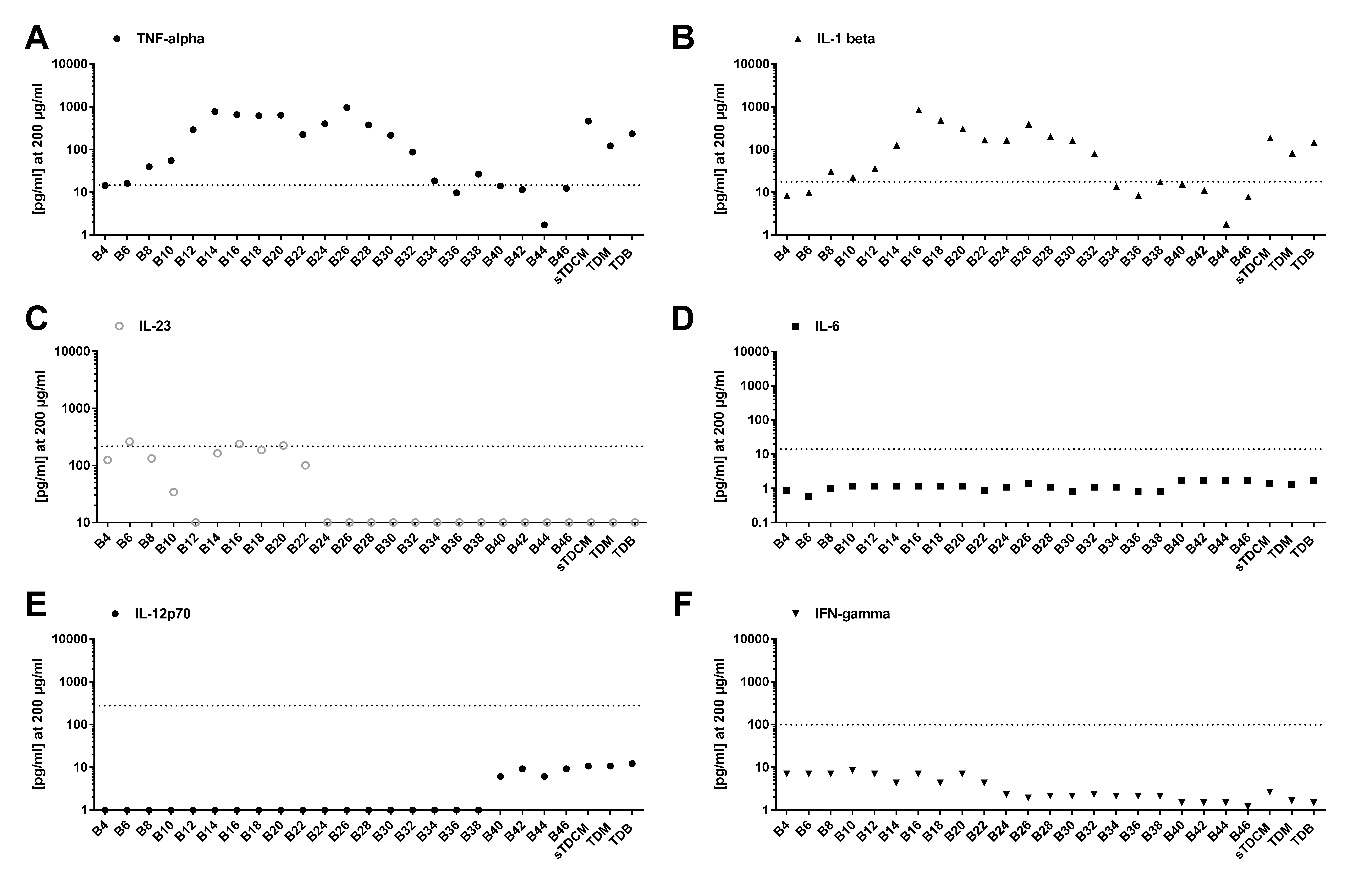


**Supplemental Figure 7.** **Cytokine production from the human macrophage cell line THP-1 in response to stimulation with synthetic αTDE compounds.** The indicated compounds were dissolved in 50% isopropanol/isooctane, serially diluted in vehicle and dried to the bottom of a tissue culture plate. THP-1 cells were applied to the compound-coated plates and incubated at 37˚C; supernatant was harvested 24 h later and analyzed for TNFα (**A**), IL-1β (**B**), IL-23 (**C**), IL-6 (**D**), IL-12p70 (**E**) and IFNɣ (**F**) cytokine production via multiplex Luminex assay. Results from the 200 µg/ml concentration are shown. Dashed lines depict the level of cytokine of the lowest standard and thus the limit of detection of a given analyte.


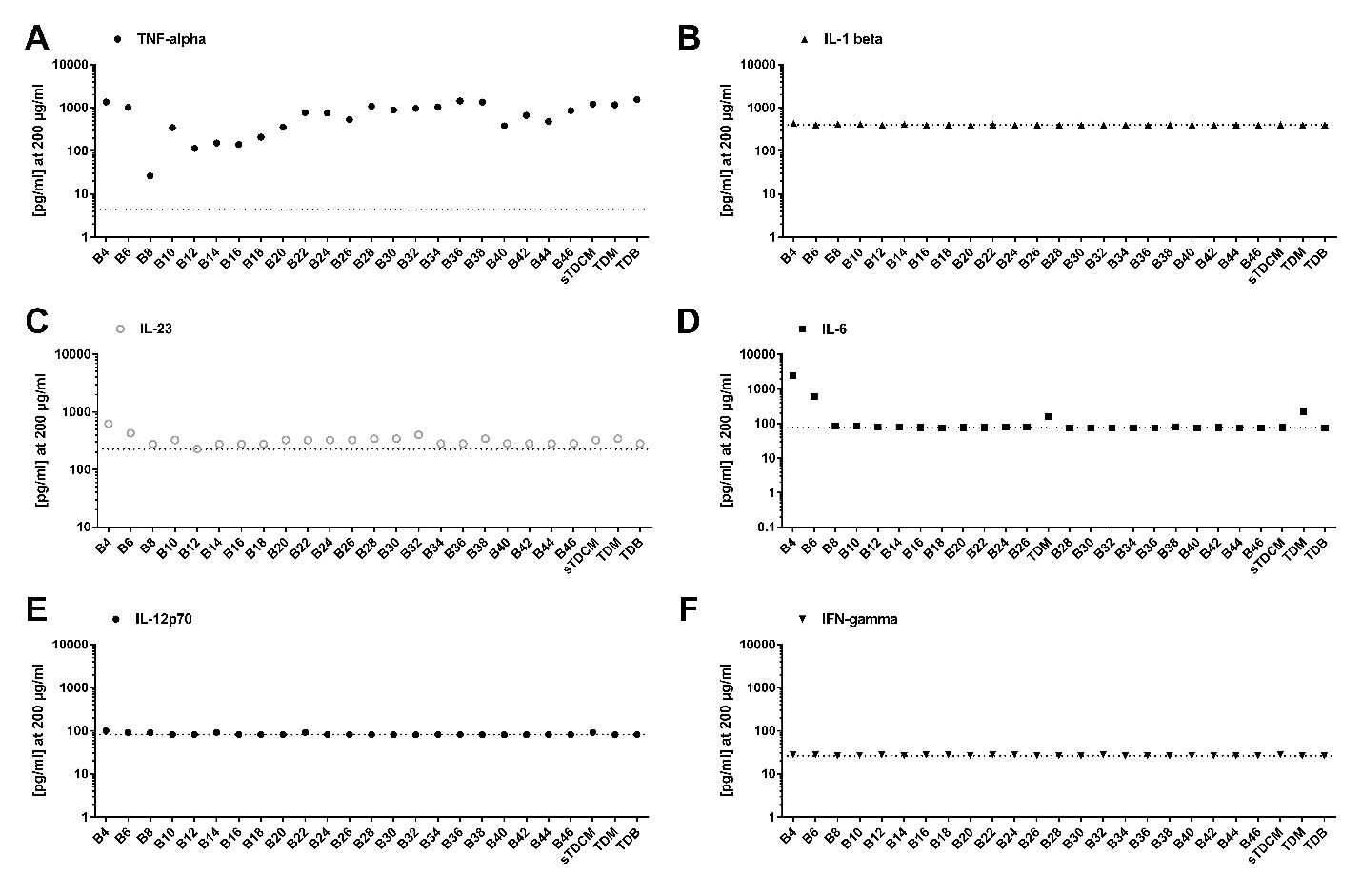


**Supplemental Figure 8.** Cytokine production from the mouse macrophage cell line RAW264.7 in response to stimulation with synthetic αTDE compounds. The indicated compounds were dissolved in 50% isopropanol/isooctane, serially diluted in vehicle and then dried to the bottom of a tissue culture plate. RAW264.7 cells were applied to the compound-coated plates and incubated at 37˚C; supernatant was harvested 24 h later and analyzed for TNFα (**A**), IL-1β (**B**), IL-23 (**C**), IL-6 (**D**), IL-12p70 (**E**) and IFNɣ (**F**) cytokine production via multiplex Luminex assay. Results from the 200 µg/ml concentration are shown. Dashed lines depict the level of cytokine of the lowest standard and thus the limit of detection of a given analyte.


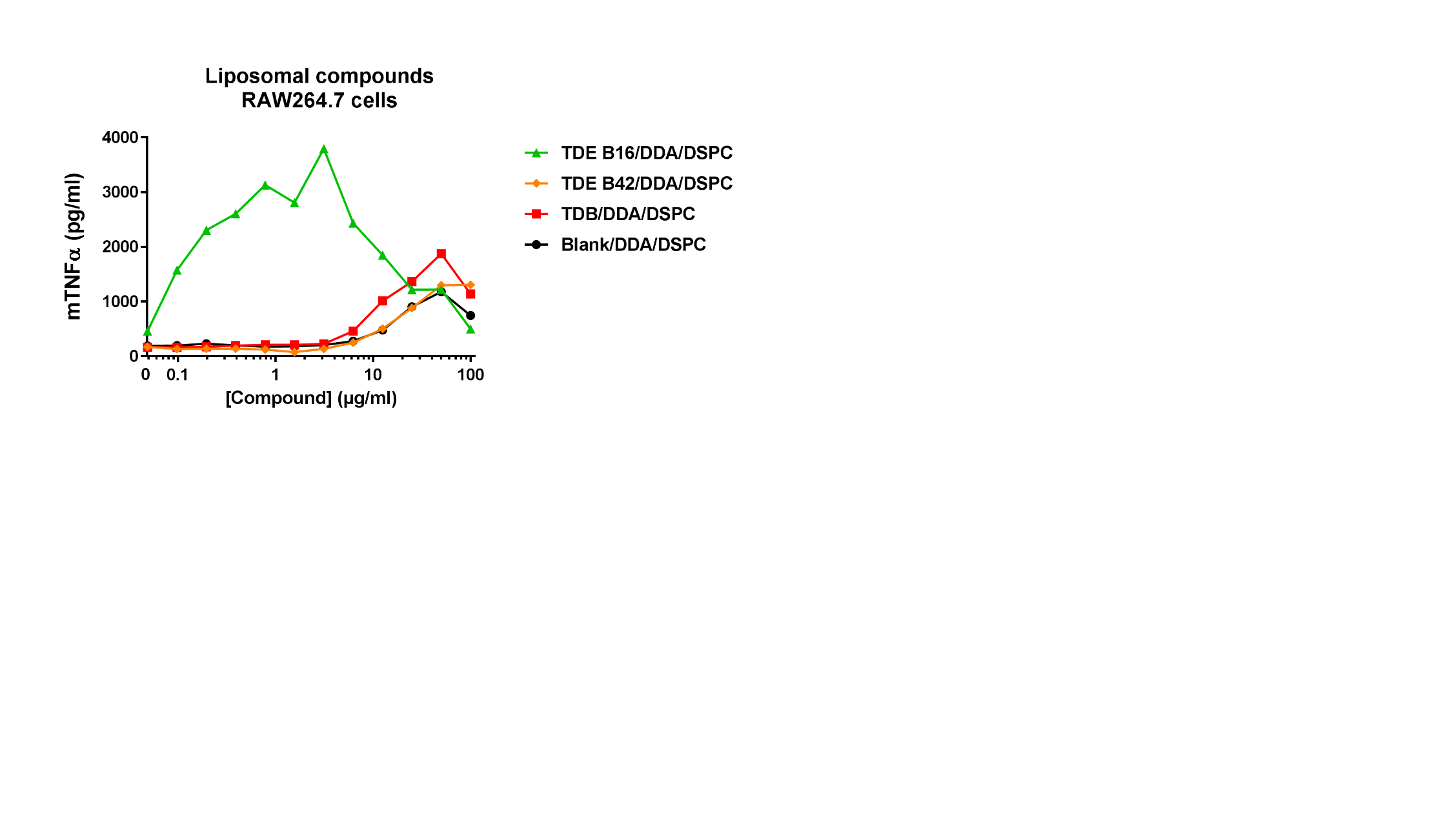


Supplemental Figure 9. *In vitro* testing of Liposome formulations used for mouse *in vivo* studies. B16 and B42, formulated in a liposome were tested for activity in RAW264.7 cells. Liposomes were made as described in the methods section and were added to RAW264.7 cultures. Blank liposomes were volume matched to compound loaded liposomes. After 24 hours, supernatants were analyzed for secreted TNFα by ELISA.


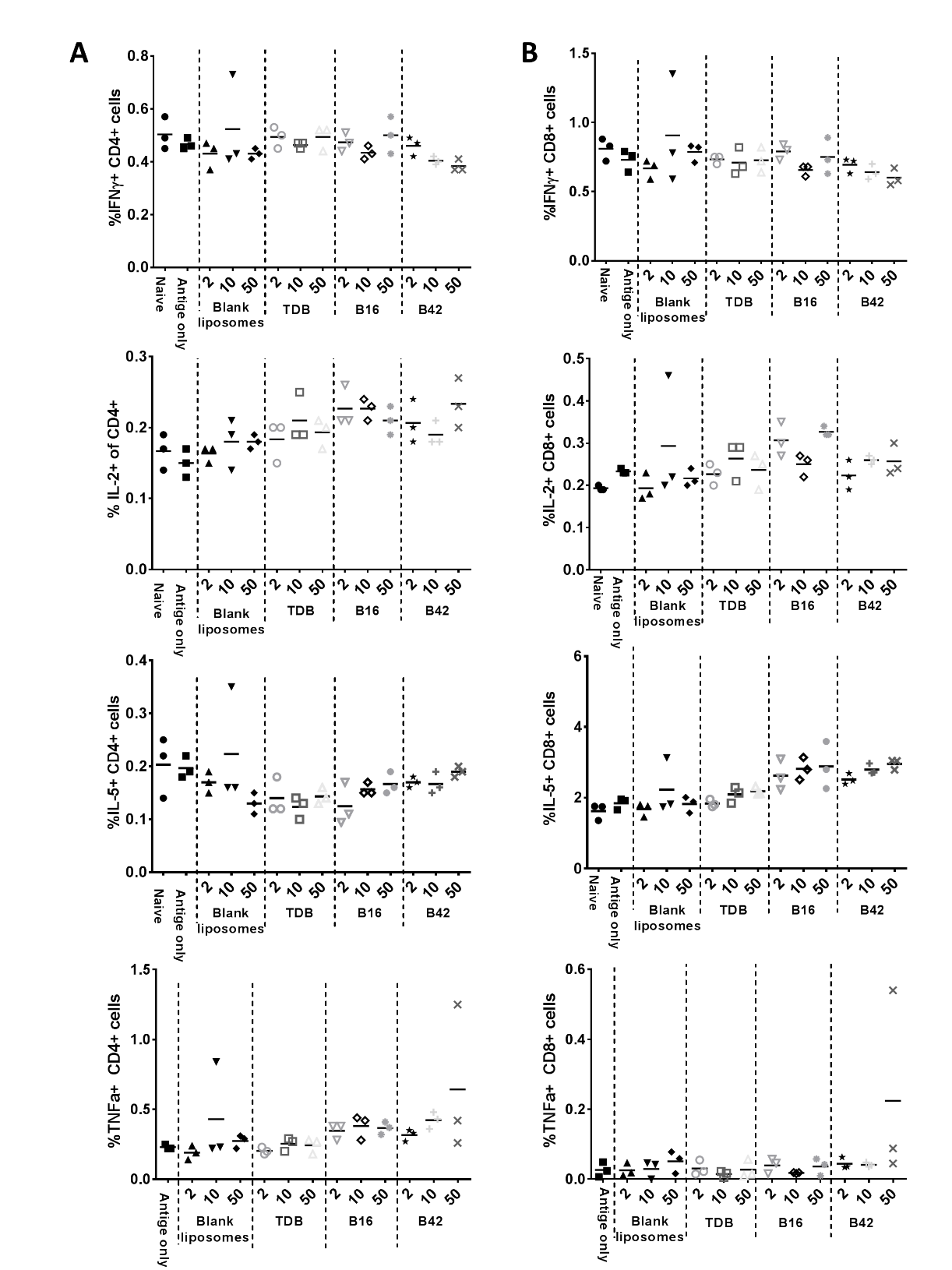


Supplemental Figure 10. Production of IFNɣ, IL-2, IL-5 or TNFα by CD4 or CD8 T cells in response to various CLR compounds. BALB/c mice, 10 per group, were immunized two times i.m. with 0.125 µg antigen plus 2, 10 or 50 nmol of the indicated adjuvant. Spleens were harvested from a subset of 3 mice per group at 5 days post-secondary vaccination. Splenocytes were restimulated with 1 µg/mL whole antigen and transport inhibitors followed by surface staining for CD3, CD4 and CD8 and intracellular cytokine staining for IFNɣ, IL5, IL2 or TNFα. Data represent percentage of live, CD3^+^/CD4^+^(A) or CD3^+^/CD8^+^ (B) T cells that are also positive for the indicated cytokine upon stimulation.


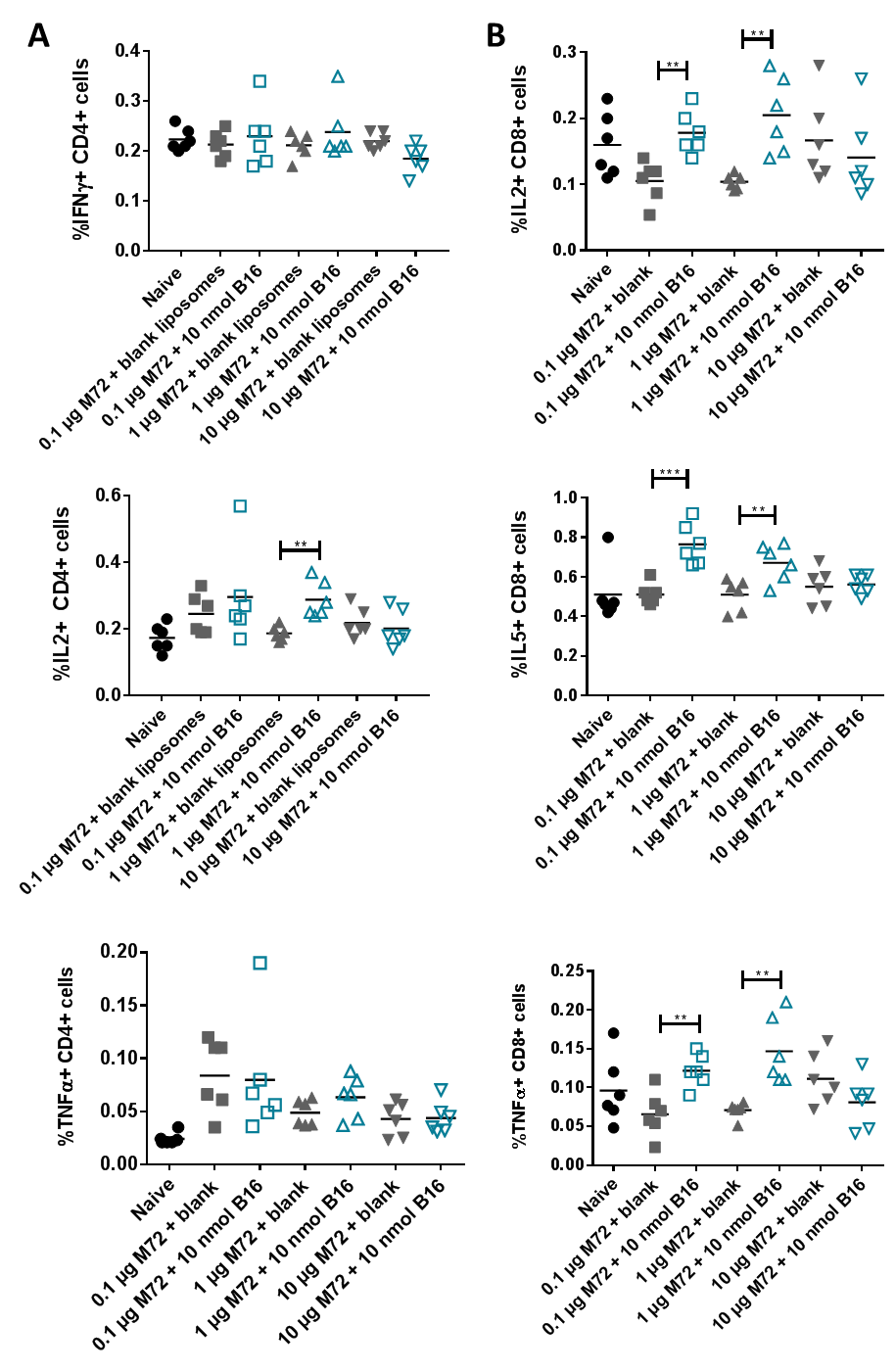


Supplemental Figure 11. Production of IFNɣ, IL-2, IL-5 or TNFα by CD4 or CD8 T cells in response to various doses of B16. BALB/c mice, 14 per group, were immunized two times, 14 days apart, i.m with 0.1, 1 or 10 μg of M72 antigen plus either blank DDA:DSPC liposome or B16 loaded (10 nmol final dose) liposomes. Spleens were harvested from 6 mice per group 5 days post-secondary vaccination and restimulated with 1 µg/mL whole antigen and transport inhibitors followed by surface staining for CD3, CD4 and CD8 and intracellular cytokine staining for IFNɣ, IL5, IL2 or TNFα. Data represent percentage of live, CD3+/CD4+ (A) or live, CD3+/CD8+ (B) T cells that are also positive for the indicated cytokine upon stimulation.. * = p<0.05, ** = p<0.01, *** = p<0.001
